# Fluorescent Polymer Platforms in Tumour Mechanobiology and Therapy Monitoring

**DOI:** 10.3390/polym18202578

**Published:** 2026-10-19

**Authors:** Xiang Chen, Yujie Li, Jiayi Chen, Wei Liu, Shengsheng Zhao, Mengting Jiang, Jing Yang, Xiaoxiao Fan, Hui Lin

**Affiliations:** 1 Department of General Surgery, Sir Run Run Shaw Hospital, School of Medicine, Zhejiang University, Hangzhou 310018, China; 2 College of Biomedical Engineering and Instrument Science, Zhejiang University, Hangzhou 310027, China; 3 Zhejiang Key Laboratory of Intelligent Medical Decision Support, Taizhou Institute of Zhejiang University, Taizhou 318000, China

**Keywords:** fluorescent polymers, tumour mechanobiology, molecular tension, local microviscosity, solid stress, polymer formulation, therapy monitoring

## Abstract

Fluorescent polymer platforms reveal different aspects of tumour mechanobiology, but the strength of the evidence behind their optical readouts varies. This narrative review distinguishes calibrated physical measurements, mechanically active therapies assessed by independent assays, and biochemical or delivery readouts. We classify polymer roles as emission generation, mechanical coupling, chemical gating, and carriage or localisation. Among the selected studies, polymer-assisted molecular rotors measured local microviscosity in mouse CT26 tumours, while fluorescent polyacrylamide inclusions enabled intravital solid-stress inference from calibrated deformation. PEG–gold tethers measured receptor tension in cancer-cell culture. Mechanically responsive fluorophores in synthetic networks remain enabling technologies without tumour validation. Our quantitative comparison covers formulation properties, optical operating conditions, calibration, and demonstrated biological settings, labelling unreported values and inapplicable metrics separately. A cholesterol-modifying polyrotaxane illustrates therapy supported by independent cellular mechanics assays; its fluorescence tracks distribution. Protease, pH, oxygen, and transcript probes provide complementary microenvironment or treatment-response information without directly measuring tissue stiffness. The evidence reviewed here establishes neither a clinically validated polymer-fluorescence biomarker of tumour mechanics nor a universal cross-platform performance ranking. Progress requires a defined physical variable and compartment, maintained formulation integrity, and evidence that the measured change informs a functional treatment outcome.

## 1. Introduction

A fluorescent polymer can reveal that a formulation has reached a tumour, but the route to an informative biological measurement continues beyond that image. The material must cross the relevant vascular and interstitial barriers, reach its intended compartment, and retain the photophysical behaviour on which the measurement depends. Tumour mechanics influences each of these steps. Compressed vessels restrict supply, collagen architecture shapes interstitial transport, and the membrane and cytoskeleton affect uptake and treatment response. These processes operate at different scales and change on different timescales [1,2,3].

Polymer chemistry offers unusual freedom to connect delivery with sensing. Conjugated chains can generate emission, hydrophilic scaffolds can carry otherwise insoluble reporters, and responsive assemblies can convert a chemical transition into an optical signal. A polymer chain can also transmit receptor-generated force to a quenched fluorophore. The same design freedom supports therapeutic functions: a fluorescent carrier may release a matrix-modifying agent or alter membrane cholesterol while its label follows distribution. The interpretation rests on how those functions are coupled [4,5,6,7].

The biological goal needs equal care. Reducing matrix-associated compression can improve vascular access, whereas increasing cancer-cell cortical stiffness can make contacted cells more susceptible to cytotoxic lymphocytes. Stress relaxation and mechanical memory add a temporal dimension that a single stiffness measurement cannot capture. A probe selected only for tumour brightness may miss the physical event most relevant to treatment. We consider the matching of polymer design, measured variable, and therapeutic question to be the central task in this field [8,9,10,11].

This narrative review examines how fluorescent polymer platforms support physical sensing, mechanically relevant delivery and therapy monitoring in tumour mechanobiology. We organise the discussion around the measurement actually demonstrated, rather than tumour brightness. We link polymer design to optical transduction, the physical variable, calibration, and spatial scale, and distinguish tumour measurements from cell-culture demonstrations, synthetic-material studies and biochemical or distribution readouts. Biochemical or distribution readouts address complementary questions and are not presented as measurements of tumour stiffness.

### 1.1. Scope and Literature Selection

We rechecked the literature on 11 October 2026 using PubMed for focused searches and Crossref for bibliographic searches and citation verification. Primary publisher and PubMed Central records were used to examine original reports. Searches combined fluorescent polymer or molecular rotor with tumour, tumor or cancer, mechanics or mechanobiology, molecular tension, microviscosity, solid stress, therapy monitoring or polymer formulation. The publication-date filter was January 2005 through September 2026. We checked accessible cited studies against their original experimental reports and retained relevant foundational studies. This selective narrative synthesis is not a systematic review or an exhaustive search and does not claim a historical screening count.

We prioritised studies in which a defined polymer component contributed to optical reporting, physical inference, tumour delivery or treatment monitoring, and the demonstrated variable and biological setting could be identified. We distinguished primary tumour measurements from cancer-cell culture, acellular materials, non-tumour imaging and mechanobiological background. Materials-only and non-tumour demonstrations remain as labelled enabling technologies and do not count as tumour validation. Genetically encoded recorders were excluded from the polymer-platform comparison. We checked numerical entries against accessible primary text and supporting information, distinguishing unverified access from an examined source that did not report a value.

### 1.2. Measurement Framework and Evidence Levels

The comparison follows polymer architecture → optical mechanism → measured variable → calibration → spatial compartment → biological interpretation. We assign four functional roles, emission generation (E), mechanical coupling (M), chemical gating (G), and carriage or localisation (C), separately from the evidence level. A platform may fulfil more than one role. The evidence categories cover physical measurement in a tumour, controlled cell-level mechanics, independently assessed mechanically active therapy, biochemical or distribution imaging, and materials-only demonstration. Localising a probe in a tumour does not, by itself, establish a physical measurement there.

**Figure 1:**
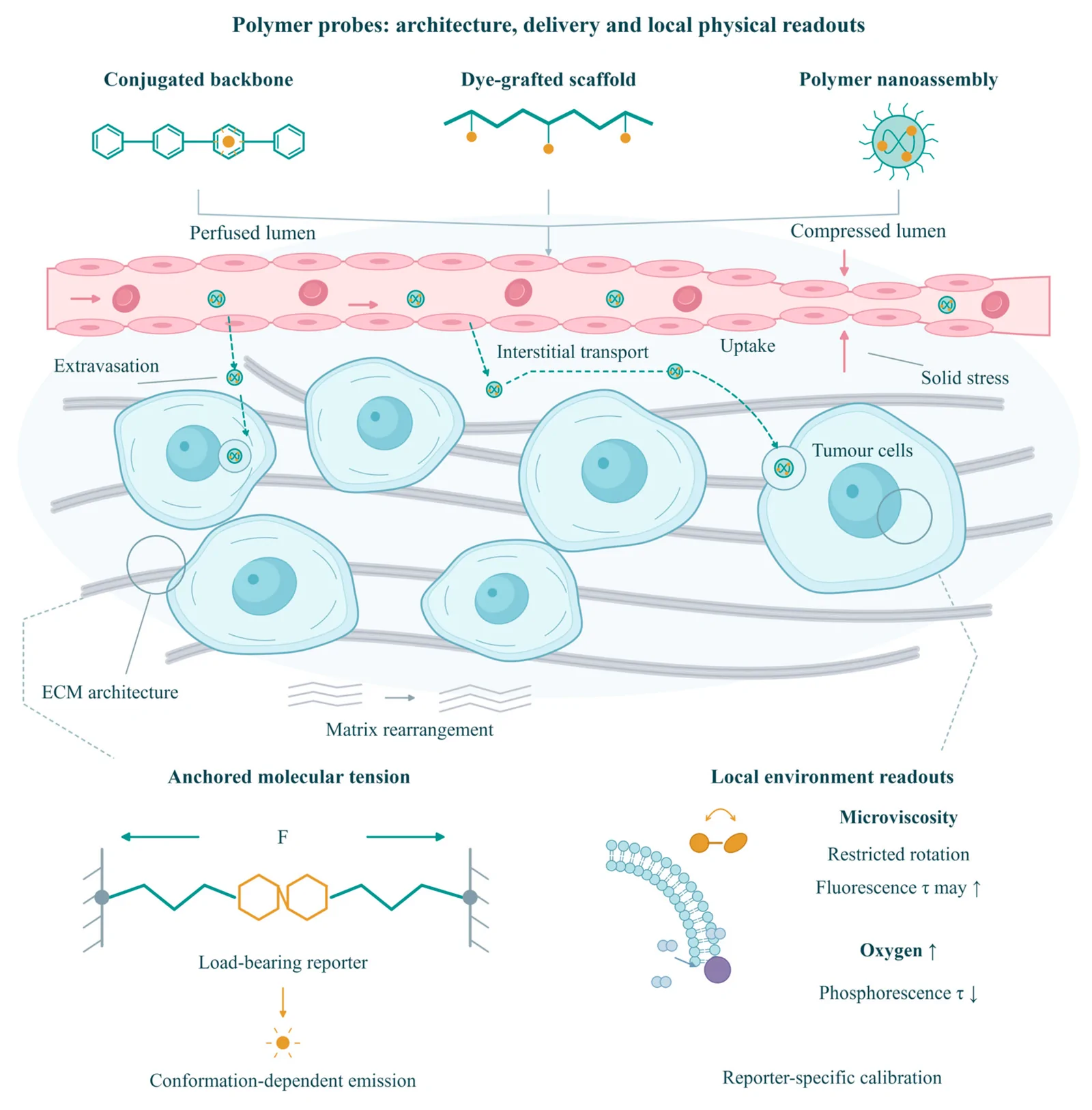
Polymer probes in a mechanically heterogeneous tumour. Conjugated chains, dye-bearing scaffolds and responsive assemblies offer distinct routes to optical reporting. Vascular exposure and tissue-level solid stress influence where a probe can reach, whereas extracellular matrix (ECM) architecture and relaxation describe the surrounding material. A force-sensitive reporter responds to tension transmitted through its molecular linkage; this quantity is distinct from bulk tissue stress. Local lifetime measurements can report microviscosity or oxygen through different reporter mechanisms and calibrations. The membrane detail locates these measurements at a cellular interface without equating their physical origins. Upward and downward arrows indicate increases and decreases, respectively; τ denotes the reporter lifetime.

To support a mechanical claim, a study must state the physical quantity, units, loading geometry and calibration range. A lifetime–viscosity relationship supports local microviscosity; a force–extension relationship supports tether tension; deformation of a calibrated elastic inclusion can support stress inference. None of these measurements automatically yields tissue stiffness. Each readout requires identification of the emitting species and sampled compartment. The complete formulation must remain valid under the measurement conditions, and an independent assay should test the biological interpretation. These general requirements are consolidated here; subsequent sections compare platforms and specify discriminating controls. Figure 1 illustrates the tumour compartments and physical quantities considered in this framework.

## 2. Tumour Mechanics and Cellular Responses

### 2.1. Matrix Architecture and Loading

The extracellular matrix provides both a load-bearing network and the spaces through which therapeutic polymers must travel. Collagen density, alignment and crosslinking alter these functions in different ways. Crosslinking can strengthen integrin-dependent signalling, while aligned fibres favour directional invasion and constrain access to tumour-cell nests. A bright polymer label along a collagen bundle may consequently indicate an accessible transport path, extracellular binding, or uptake by adjacent stromal cells. Resolving its position relative to fibres and cell boundaries is the first step towards distinguishing these possibilities [12,13,14].

Matrix stiffness, solid stress and interstitial fluid pressure also describe different physical quantities. Stiffness is the resistance to deformation under specified loading conditions; solid stress reflects forces borne by the solid constituents, including compression of vessels; fluid pressure belongs to the interstitial fluid phase. Stress relaxation adds a temporal dimension: a matrix can resist an initial deformation yet progressively release that resistance. Collagen images provide architectural information, but neither an elastic modulus nor a local stress follows directly from fluorescence intensity. Probe interpretation must retain the relevant length scale, loading history, and sampling compartment [1,2,15].

Fan and colleagues found that stress relaxation and viscous dissipation could change without a corresponding stiffness increase in the pre-cirrhotic liver. Independently tunable alginate–basement-membrane networks linked these changes to integrin β1, tensin 1, and YAP-associated growth and invasion. These findings give a biological reason to measure relaxation separately from stiffness [9].

Heterogeneity within the cellular compartment further complicates a tissue-level description. Fuhs and colleagues examined human breast and cervical carcinoma explants across multiple length scales and found broad distributions of cell rigidity, including soft cells within mechanically stable tumour tissue. Their microscopy and rheological measurements identified coexisting mobile and arrested regions. Thus, a stiff tumour need not contain uniformly stiff cancer cells. For a fluorescent polymer probe, averaging across a tissue section can conceal the population that controls invasion or immune susceptibility. Spatially registered measurements should retain the distribution of responses and identify the cells contributing to its extremes. This also changes the interpretation of treatment: a shift in the mean signal may reflect altered cell composition or selective depletion, rather than a comparable mechanical change in every surviving cell [16].

### 2.2. Cellular Mechanosignalling and Membrane Mechanics

Integrin engagement, actomyosin, and nuclear coupling translate mechanical input into signalling through YAP and TAZ. Nuclear YAP localisation reflects a downstream state, while an anchored tension reporter samples receptor loading. Because they answer different questions, these measurements should be assessed separately rather than pooled as polymer sensor performance [5,17,18,19].

At the plasma membrane, cortical stiffness, bilayer tension, membrane–cortex coupling and lipid organisation may change together without being interchangeable. Their distinction matters for both nanoparticle uptake and immune attack. Cholesterol depletion, for example, stiffened cancer cells and increased their susceptibility to cytotoxic T cells in preclinical experiments. A membrane-localised fluorophore may respond to the accompanying lipid reorganisation, whereas indentation measures the resistance of the cell to an applied load. Agreement between these readouts must be established in the same perturbation and cell population [8].

Caveolae can flatten to buffer membrane loading, while lipid chemistry can alter their organisation. Caveolin-1 abundance or lipid peroxidation thus helps explain the mechanism but does not provide a numerical membrane-tension measurement [20,21,22].

### 2.3. Mechanical Memory and the Timing of Measurement

Matrix exposure can leave persistent cellular programmes. In melanoma, Maiques and colleagues linked matrix sensing at the invasive front to subsequent invasion and metastasis, including sustained STAT3 signalling after removal from the inducing environment [11]. These findings motivate repeated physical measurements before and after treatment. They provide biological background, without demonstrating a fluorescent-polymer sensor.

Mechanical history also affects lymphocytes. Postat and colleagues associated stronger mechanical input with changes in nuclear-envelope composition and a tissue-resident-memory transcriptional programme in activated CD8-positive T cells [23]. Therapy studies should therefore retain information on cell identity and exposure history.

Table 1 relates these biological scales to the physical quantities accessible to fluorescence and complementary measurements.

## 3. Polymer Architecture and Responsive Chemistry

### 3.1. Emissive Conjugated Backbones

Hong and colleagues dispersed an emissive donor–acceptor copolymer (pDA) with phospholipid–PEG and demonstrated rapid NIR-II arterial imaging in mice [4]. The conjugated polymer generates the signal; the shell supports dispersion. The experiment demonstrates temporal vascular imaging capability without measuring tumour solid stress or stiffness. We discuss its proposed use after tumour decompression only as a future experiment in Section 8.2.

Equal polymer mass is an incomplete basis for comparing dose. For a known linear polymer, chain molarity depends on number-average molecular weight, not weight-average molecular weight. Dispersity remains relevant when chain length affects loading or response. Particle number also depends on aggregation number, size distribution, and formulation integrity. Chromophore amount depends on measured content and whether the chromophore remains polymer-associated. Equal injected masses can therefore produce unequal chain, particle, and chromophore exposures. The denominator should follow the question: chain or ligand density for anchored tension probes, reporter molarity for calibration, particle number for uptake, and active-payload mass for therapy. Where measurable, these quantities should be reported together [3,4,5].

Optical comparisons require a second set of controls. Even at equal chromophore amounts, detected brightness can vary with absorbed excitation photons, quantum yield, acquisition geometry, spectral filters, and tissue depth. Matching absorbance can help compare photophysics, yet it need not preserve equal biological dose. A ratio or lifetime can reduce some concentration dependence; neither corrects aggregation, mixed compartments or differential clearance. Studies should report the chosen normalisation, available alternative exposure measures and a concentration series. No single denominator makes different polymer architectures universally comparable. Stability must also be separated into colloidal size, chemical integrity, cargo retention and photophysical persistence, with medium, temperature and duration specified [3,4,6].

Backbone planarisation presents a central compromise in near-infrared polymer design. Extending electronic conjugation improves light absorption and can shift emission towards longer wavelengths, but close interchain packing can introduce non-radiative decay. Su and colleagues addressed this balance by varying the proportion of planar and twisted segments in phenothiazine–benzobisthiadiazole-based copolymers. Their series linked monomer composition to absorption, aggregation-dependent emission, and photothermal conversion. An intermediate composition retained aggregation-induced emission while absorbing more strongly at the irradiation wavelength than the fully twisted analogue, supporting NIR-II imaging and photothermal treatment in a mouse tumour model. This comparison identifies a practical optimisation target: brightness at the intended excitation wavelength, together with the required therapeutic energy conversion, rather than the fluorescence quantum yield alone [33].

Hsu and colleagues obtained 1064–1100-nm emission from TADF-derived units in donor–acceptor polymer dots, although the final particles emitted promptly rather than with a delay. Vascular and skeletal imaging demonstrated photophysical and anatomical capabilities without tumour-mechanics validation [34]. We therefore include this study as an enabling optical comparison rather than a mechanical sensor.

Covalently incorporating a chromophore offers another route to signal stability. Lee and colleagues reported the cyanine-containing polymer KR-NIR-P, which was more resistant to photobleaching than indocyanine green under the tested irradiation conditions but had a lower quantum yield in methanol. Their footpad-to-lymph-node experiment examined lymphatic transport; it did not test intravenous tumour access [35]. Photostability and quantum yield remain separate measures of performance.

### 3.2. Covalent Dye Conjugates and Polymeric Carriers

Covalent attachment to a hydrophilic polymer allows control over average dye loading and linker chemistry and can slow the loss of an otherwise unbound fluorophore. Cleavage or polymer degradation may subsequently release the label. The species present at the imaging site—intact conjugate, free dye, or a fluorescent degradation fragment—then determines its cellular distribution and clearance.

Shimolina and colleagues used amphiphilic polymer brushes to deliver hydrophobic BODIPY1 for FLIM of CT26 tumour microviscosity [6]. The brushes mainly provide solubilisation and localisation; viscosity sensitivity comes from the rotor. The polymer-delivered rotor predominantly sampled collagen-associated extracellular regions, while the water-soluble BODIPY2 comparator sampled tumour cells. The reporters therefore cannot be treated as measurements of the same compartment. Formulation-dependent aggregation invalidated viscosity readouts in some samples. This illustrates how a carrier can affect sensing validity even when it does not act as the transducer.

In an activatable conjugate, dye loading, quencher proximity, substrate accessibility and linker stability determine how efficiently a biochemical event produces fluorescence. Characterisation before and after activation must identify both the emitting product and its location. Once released, a bright small-molecule product may no longer trace the parent polymer.

Fluorescence can also emerge from an aliphatic polymer without an extended aromatic chromophore. Zhou and colleagues synthesised PEG-capped polyureas from lysine- and cystine-derived monomers. Hydrogen bonding brought carbonyl-containing groups into close proximity, producing intrinsic emission attributed to clustering. Chain length and conformation altered both the assembled morphology and fluorescence. Perturbing hydrogen bonding changed vesicle or nanotube dimensions together with the optical spectrum, while monomer controls remained weakly emissive. This behaviour differs from attaching an independently fluorescent dye to an otherwise passive carrier: the emitting environment is generated by the assembled polymer itself [36].

### 3.3. Responsive Assemblies and Supramolecular Polymers

Amphiphilic polymers couple protonation, cleavage, and intermolecular association to changes in assembly. Close packing can quench fluorophores, with disassembly restoring emission; other chromophores emit more strongly when aggregation restricts their motion. These responses impose different requirements on reporter position and core mobility. Nanoparticle morphology alone reveals little about the underlying photophysics.

Multicomponent assemblies permit more controlled comparisons. Mixing chemically compatible reporter-bearing and reporter-free chains can vary fluorescence density at approximately constant total polymer concentration, provided that co-assembly and homogeneous mixing are verified. Modifying the hydrophobic block can change core association alongside particle size, protein interactions, and uptake. All of these changes bear on a structure–signal relationship. Programmable polymer libraries encode chemical inputs in carrier behaviour, although a logic-gated release system qualifies as a fluorescent sensor only when its optical output has been experimentally linked to the intended input [37].

Pan and colleagues varied non-ionisable comonomers in a tertiary-amine-containing methacrylate core while retaining a PEG corona. Core hydrophobicity altered nanoparticle–cell interactions and the fluorescence generated after pH-dependent activation. Optical performance depended on both the responsive transition and the cellular access conferred by the core composition [28].

Polyrotaxanes can combine imaging and therapeutic components within a different supramolecular architecture. Luo and colleagues incorporated methyl-β-cyclodextrin units into a diselenide-containing formulation with fluorescent IR783 end groups; the formulation could also carry the stimulator of interferon genes (STING) agonist diABZI. The threaded architecture associates the fluorophore with cholesterol-modifying chemistry and provides a distinct site for the immune payload. The fate of each component after cleavage determines how imaging and therapeutic functions remain coupled [7].

The non-emissive surface component can determine whether those optical properties are accessible in a biological model. Karabacak and colleagues formed near-infrared dots from a neutral thiophene–benzobisthiadiazole donor–acceptor–donor polymer using cetyltrimethylammonium bromide. The surfactant supplied the strongly positive surface charge, while the conjugated backbone supplied emission near 860 nm. The reported hydrodynamic radius was approximately 12 nm. Imaging in three tumour-spheroid models revealed fluorescence within central regions and overlap with mitochondrial staining. This is evidence for penetration and localisation in those models, rather than for systemic tumour selectivity. It also distinguishes two design variables that are often bundled together: polymer chemistry establishes the optical response, whereas the formulation can dominate the initial interaction with cells and extracellular material [38].

Self-immolative poly(thiocarbamate) links hydrogen-sulphide recognition to depolymerisation and chemical amplification [39]. The polymer thus acts as a chemical gate, and its formulations report a reaction network rather than tissue mechanics.

### 3.4. Polymer Chains as Mechanical Elements

Force sensing requires the reporter to lie within a load path that couples deformation to an optical response. A polymer may supply the extensible element or transmit force to a mechanically responsive chromophore. Labelling a deformable particle is insufficient unless that coupling has been characterised.

Liu and colleagues placed a PEG tether between a fluorescent integrin ligand and a gold nanoparticle. Receptor-generated tension extended the tether, increasing fluorophore–particle separation and reducing nanometal surface energy-transfer quenching. PEG served as the molecular spring and gold as the quencher. Measurements at this living-cell–substrate interface relied on calibrated chain extension and stable surface anchoring; a freely circulating formulation would require a different measurement geometry [5].

We use four polymer roles throughout: E, the polymer generates emission; M, it provides an essential mechanical load path or a calibrated deformable body; G, its chemical transition gates optical activation; and C, it carries, stabilises or localises an independently sensing reporter. These roles can coexist. A brush carrying a rotor is mainly C, and a PEG spring is M. A pH-responsive fluorescent micelle is G plus C; an emissive conjugated backbone is E. A fluorescent elastic inclusion belongs to M because its characterised deformation enables stress inference, even though the fluorophore serves only as a fiducial marker. Table 2 assigns polymer role separately from biological evidence level.

### 3.5. Comparative Strengths and Practical Limits

The rotor formulation and elastic inclusions provide calibrated physical measurements in living mouse tumours, but of different quantities and compartments. PEG–gold tethers have a defined pN response at an anchored cancer-cell interface, without animal-tumour validation. Synthetic mechanofluorescent networks have explicit load coupling but remain outside tumour models. In the polyrotaxane study, a mechanically active intervention was supported by independent cell-mechanics and treatment assays; the fluorescent channel tracked distribution. Biochemical reporters can monitor therapy-associated pH, transcripts, proteases, or oxygen-sensitive decay. These readouts cannot be ranked against tension or solid-stress sensors using a common mechanical sensitivity.

The table therefore compares the demonstrated task, formulation, calibration and experimental access, rather than assigning a universal winner. Molecular weight characterises polymer chains, DLS size characterises dispersed particles, and loading efficiency describes recovery or incorporation under a specified definition. Carrier content, dye substitution and encapsulation efficiency are not interchangeable. Any numerical advantage applies to the stated medium, irradiation, dose, model, and acquisition time. Differences between papers do not constitute controlled head-to-head performance tests.

### 3.6. Structural Control of Optical Output

**Figure 2:**
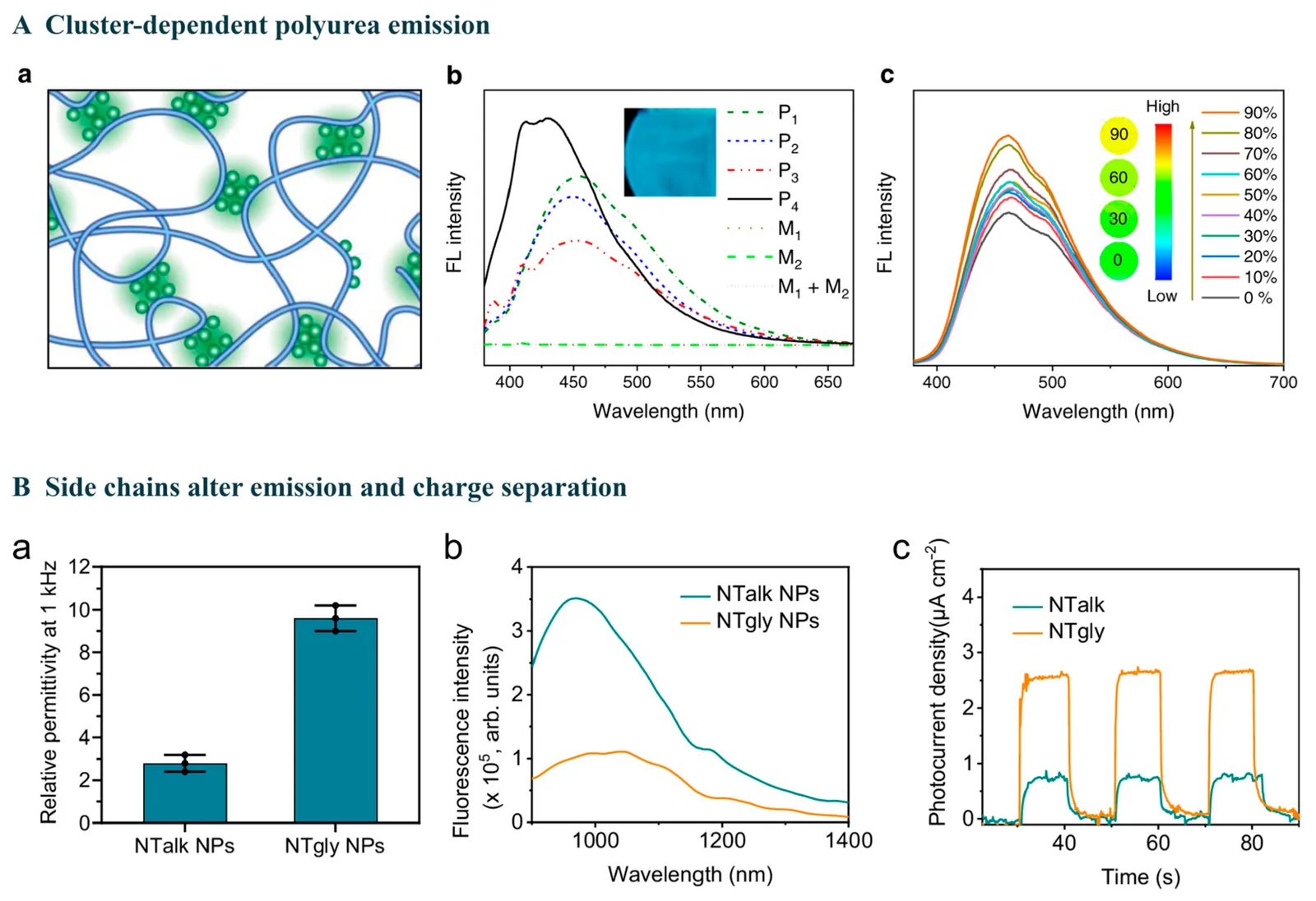
Polymer organisation controls the balance of optical outputs. (**A**) Proposed carbonyl-cluster emission mechanism of polyureas, fluorescence spectra of polymer assemblies and monomer controls, and aggregation-dependent emission as the chloroform fraction in DMF increases (original panels (**a**–**c**)). (**B**) Relative permittivity of dry polymer films at 1 kHz, nanoparticle fluorescence spectra under 808-nm excitation, and transient photocurrent of polymer films, comparing alkyl-substituted NTalk with glycol-substituted NTgly (original panels (**a**–**c**)). Permittivity bars show mean ± SD of three measurements, as documented in the source data; the source specifies *n* = 3 independent samples and mean ± SD for the fluorescence spectra. Glycol substitution increases permittivity and photocurrent while reducing fluorescence. Selected panels from Zhou et al. [36] and Tang et al. [44].

Figure 2 contrasts two structural routes to optical control: cluster-dependent emission in an aliphatic polyurea and side-chain-dependent energy conversion in a conjugated photosensitiser. In the polyurea, hydrogen bonding and chain conformation generate the emitting clusters (Section 3.2). In the photosensitiser, glycol side chains increase permittivity and photocurrent while reducing fluorescence, a trade-off discussed further in Section 8.2. Both examples show that polymer organisation, not only the chromophore, determines the optical output.

## 4. Probe Transport Through the Tumour

**Figure 3:**
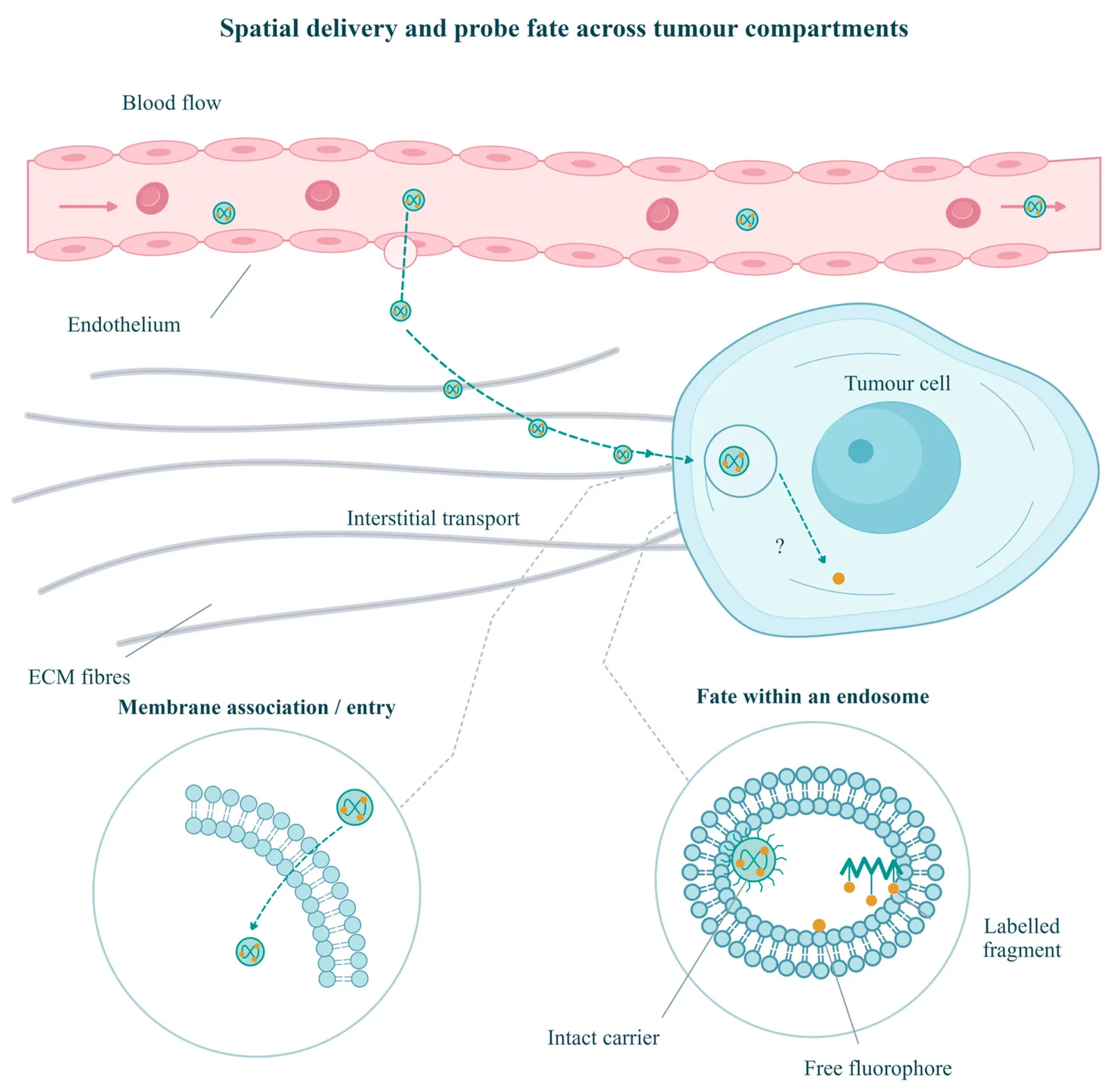
Spatial delivery and probe fate across tumour compartments. A circulating polymer probe may cross the endothelium, move through the fibrous extracellular matrix (ECM), associate with a tumour-cell membrane and enter an endosomal compartment. Dashed arrows denote conditional routes rather than measured flux or assured delivery. The membrane enlargement represents association or entry without prescribing one uptake mechanism. Within the endosomal bilayer, an intact carrier, a dye-bearing polymer fragment and a free fluorophore illustrate possible emitting species whose distributions may differ. They are alternatives to distinguish experimentally, rather than a necessary degradation sequence. Orange dots denote fluorophores, whether polymer-associated or free, according to the illustrated probe state.

Figure 3 follows a fluorescent formulation across vascular, interstitial and cellular compartments. Each transition changes the possible identity and interpretation of the emitting species, so the transport question must be defined before intensity is compared.

A well-characterised optical response is useful only in the compartment the probe reaches. Polymer mechanics, geometry and assembly influence that access, sometimes favouring one delivery step at the expense of another. For fluorescent probes, these transport properties also determine which parts of a heterogeneous tumour contribute to the measurement [3,45].

### 4.1. Deformability and Geometry

Anselmo and colleagues prepared PEG hydrogel nanoparticles of approximately 200 nm with tunable elasticity. Softer particles, exemplified by a modulus of 10 kPa, circulated longer than particles with a modulus of 3000 kPa. In vitro, softer formulations showed lower uptake by J774 macrophages, bEnd.3 endothelial cells and 4T1 cancer cells. Longer circulation and efficient cellular entry thus favoured different mechanical properties in these experiments. For an extracellular reporter, reduced uptake may help preserve access to the intended compartment; an intracellular sensor must overcome the same uptake barrier. These implications should guide formulation testing rather than establish a universal preferred modulus [46].

The dependence on deformability changes with scale and biological interface. Red-blood-cell-sized hydrogel particles in Merkel and colleagues’ study circulated longer when their modulus decreased, with reduced trapping in organs such as the lung. That result concerns passage of micrometre-scale objects through the circulation and should not be treated as a universal rule for nanoprobes entering tumours [47]. At the nanoscale, Li and colleagues compared lipid-coated hydrogel cores with different crosslink densities, with hydrogel moduli estimated from corresponding bulk gels, alongside softer liposomes and stiffer polymer cores. Circulation lifetime varied non-monotonically across the tested elasticity range. Apolipoprotein A-I enrichment in the protein corona correlated with prolonged circulation, and antibody-shielding experiments supported its contribution. The implication is that polymer mechanics can alter exposure indirectly by changing the acquired biological interface. A modulus measured before injection therefore describes only part of the delivery system; fluorescence-retention studies benefit from corresponding measurements of corona composition, particle integrity, and uptake by relevant blood and tissue cells [48].

Geometry introduces another dependence. Using polystyrene targets and alveolar macrophages, Champion and Mitragotri showed that local shape at the first contact determined whether engulfment began, whereas target size could limit its completion. The study concerns phagocytosis of model particles, not a general ranking of nanoprobe shapes for tumour penetration. It nevertheless shows why an equivalent diameter alone incompletely describes the interface presented to a cell [49].

### 4.2. Endothelial Crossing and Interstitial Distribution

Evidence for active endothelial transport also argues against predicting access from pore size alone. Sindhwani and colleagues combined imaging and mechanistic analyses to identify transendothelial entry as a major route in the tumour models and nanoparticles they examined. Their findings challenge an exclusively paracellular account of accumulation, while leaving route dependence on vascular phenotype and formulation unresolved [50]. For fluorescent polymers, the discriminating experiment must locate the label during passage: endothelial association, intracellular vesicles, and appearance beyond the vessel are successive observations. More fluorescence in endothelial cells can indicate increased capture without establishing productive delivery into the interstitium.

Endothelial passage is an active cellular process as well as a permeability problem. Wu and colleagues identified macropinocytosis as a predominant route across tumour endothelium in their experimental systems: membrane ruffles captured particles, vesicles transported them through cells, and release delivered them to the interstitium. Particle size changed the contributions of different entry pathways. This evidence argues against interpreting tumour accumulation solely through the dimensions of endothelial gaps, while leaving the dominant route dependent on formulation and vascular context [51].

Surface chemistry can exploit a more specific interaction. Fucoidan-based carriers targeted endothelial P-selectin and crossed the blood–brain barrier through caveolin-1-dependent transcytosis in a Sonic hedgehog medulloblastoma mouse model; irradiation enhanced delivery. Here, a polysaccharide helped define the biological interface, and vascular activation contributed to performance. The result supports a route for that carrier–tumour combination, rather than unrestricted brain access [52].

Annexin A2 pre-coating provides a complementary comparison. It enhanced delivery of lipid-coated mesoporous silica particles and liposomes in breast and pancreatic tumour models through an interaction involving endothelial α5β1 integrin. Responses differed with vascular integrin expression. Although these carriers are not themselves polymer-fluorescence sensors, they show how surface recognition can determine access to the tissue being imaged [53].

Crossing a vessel is only the first spatial transition. Collagen organisation, accessible pores and binding sites influence subsequent distribution, while vascular compression limits the supply entering the region. A probe intended to assess matrix remodelling should therefore be localised relative to perfused vessels, stromal structures and recipient cells. Increased fluorescence near vessels may reflect greater supply without deeper penetration. Conversely, wider extravascular distribution could follow a change in binding or assembly as well as a change in matrix mechanics. Spatial profiles, perfusion measurements and independent mechanical assays help identify which barrier an intervention has altered [1,13,54].

**Figure 4:**
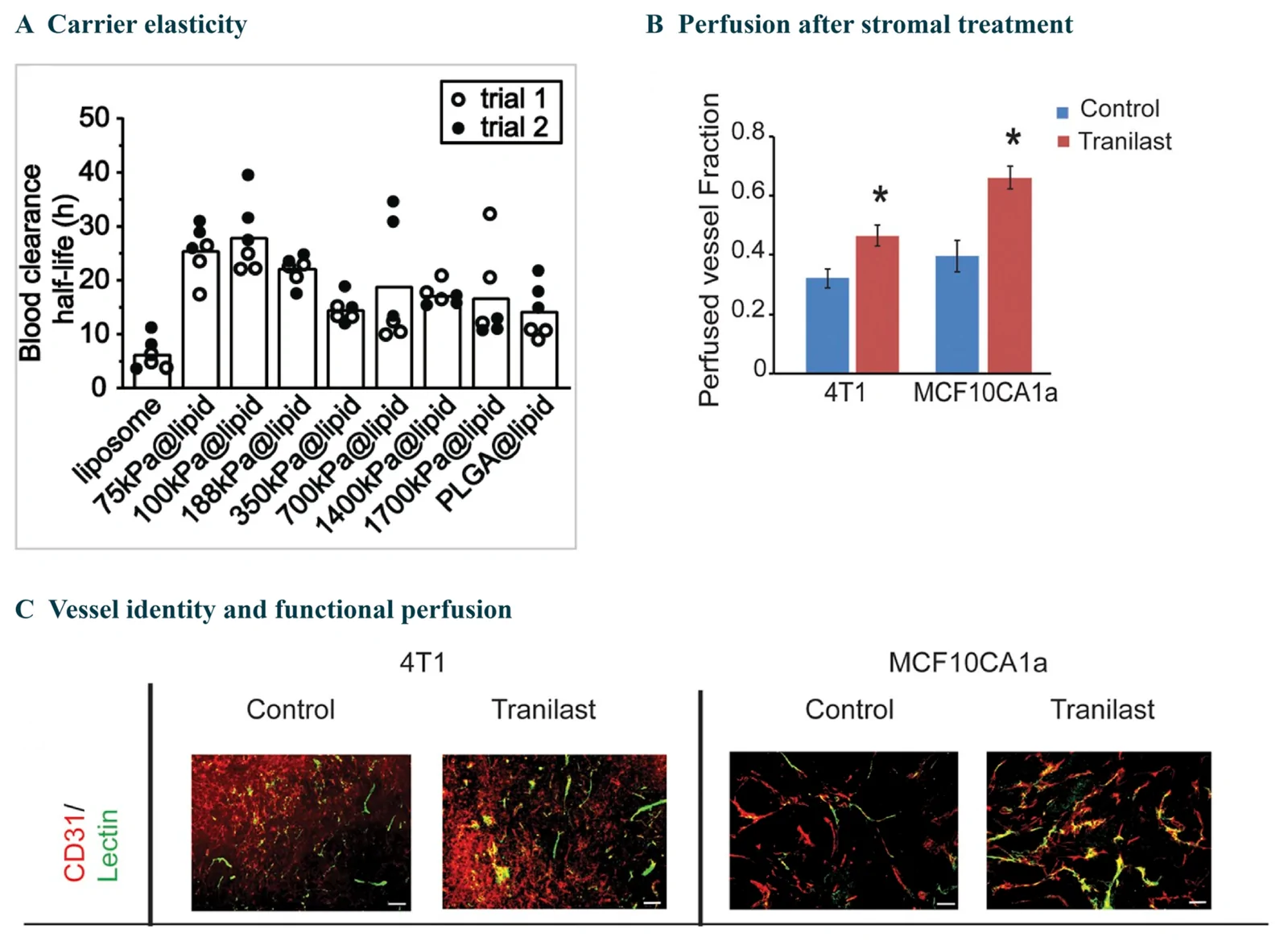
Circulation and perfusion impose distinct constraints on tumour delivery. (**A**) Blood-clearance half-lives for lipid-coated hydrogel cores of differing elasticity and liposome/PLGA controls; bars average two independent trials, each with three biologically independent mice. Hydrogel moduli were estimated from corresponding bulk gels. (**B**,**C**) Tranilast-associated changes in perfused-vessel fraction and representative CD31/lectin images in 4T1 and MCF10CA1a tumours. Red denotes CD31, green denotes lectin, and overlap identifies perfused vessels. Scale bars: 100 μm. Perfusion data are means ± SE; the source reports *n* = 6–8 for these comparisons, with five sections analysed per tumour. Student’s *t*-test: *p* = 0.027 for 4T1 and *p* < 0.001 for MCF10CA1a; asterisks denote *p* < 0.05.

Figure 4 juxtaposes carrier-dependent circulation with stromal-treatment-dependent perfusion. These measurements address different delivery constraints; neither alone establishes productive entry into the intended tumour cells.

### 4.3. Responsive Assembly and Intracellular Access

Responsive polymers can change the size presented to successive barriers. Li and colleagues used an amphiphilic polymer containing ionisable tertiary amines to assemble platinum-prodrug-conjugated PAMAM dendrimers into superstructures of approximately 80 nm. At mildly acidic pH, around 6.5–7.0, these structures dissociated into dendrimer units smaller than 10 nm. Comparison with pH-insensitive structures of similar size, charge and composition supported improved penetration in multicellular spheroids and BxPC-3 pancreatic tumour models. This is a chemically triggered transport strategy; the assembly transition is not a direct response to force [55].

The recipient cell can determine uptake as strongly as carrier design. In CAL51 triple-negative breast cancer cells, Cassani and colleagues linked YAP depletion to changes in membrane organisation, mechanics and endocytic programmes, with increased uptake of the tested nanoparticles and doxorubicin-loaded liposomes. Substrate comparisons were particularly informative: fibronectin restored nuclear YAP on a soft surface and opposed the increase in uptake observed with collagen. A nominal substrate modulus therefore failed to predict the response independently of ligand chemistry. These experiments support stratifying cells by mechanosignalling state when evaluating fluorescent carriers. They also favour measuring intracellular drug distribution alongside carrier association, because a treatment that changes endocytosis can alter both exposure and the optical readout used to assess its effect [56].

## 5. Optical Measurements and Their Mechanical Evidence

**Figure 5:**
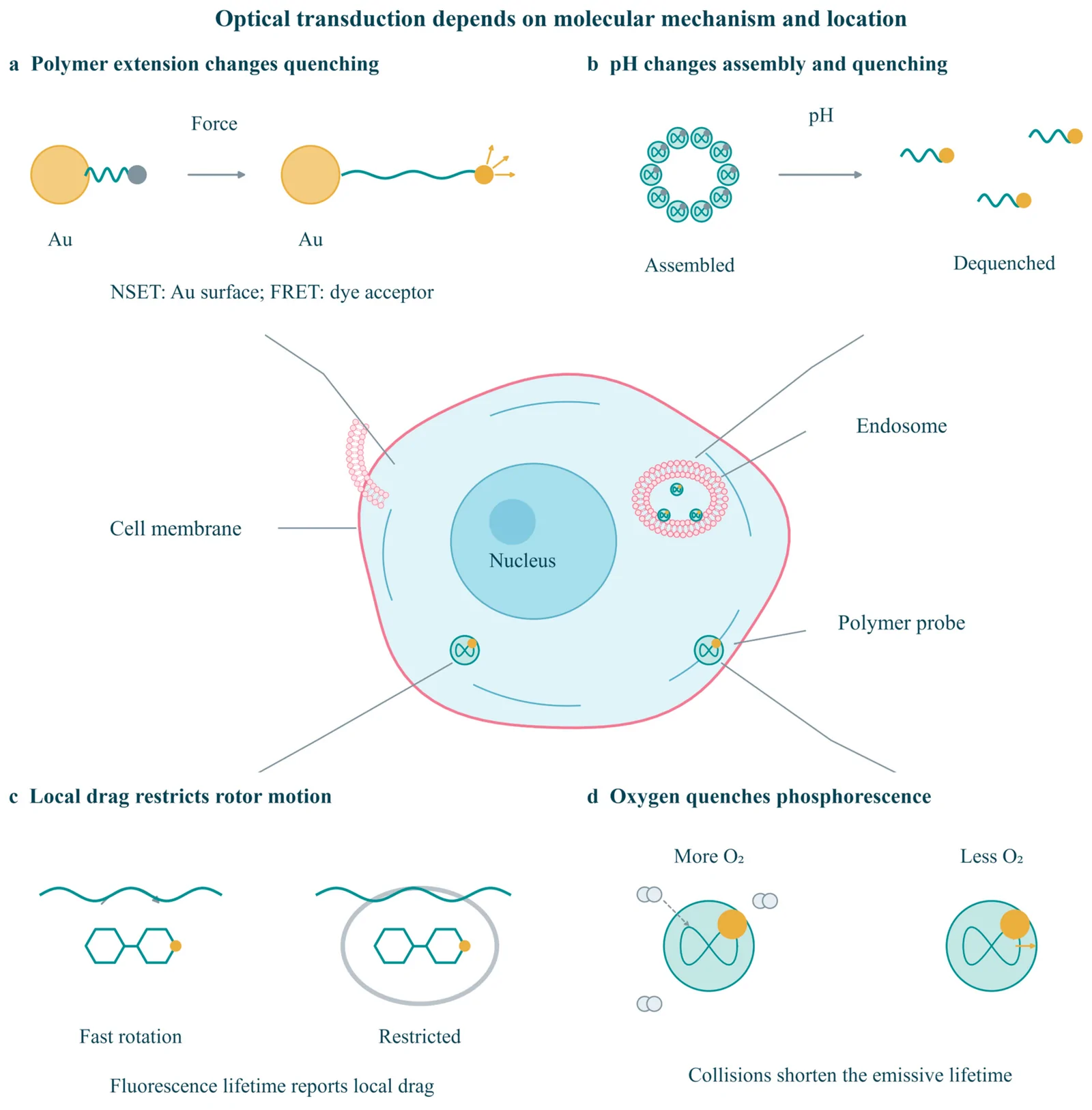
Molecular mechanisms underlying polymer-associated optical measurements. The central cell locates illustrative membrane, endosomal, and intracellular measurements; it does not represent a single formulation that combines all four functions. (**a**) Extension of a polymer tether separates a fluorophore from a gold surface and can relieve nanometal surface energy-transfer (NSET) quenching. Förster resonance energy transfer (FRET) instead involves a donor–acceptor pair and requires a different distance calibration. Force inference additionally requires known tether attachment and force–extension behaviour. (**b**) An example of pH-responsive disassembly separates quenched chromophores and increases emission; the transition depends on polymer chemistry. (**c**) Restriction of molecular-rotor motion alters fluorescence lifetime. The generic rotor motif illustrates intramolecular motion rather than a specific chemical structure; calibrated lifetime reports local microviscosity, not tissue elastic modulus. (**d**) Oxygen collisions quench an emissive triplet state and can shorten the phosphorescence lifetime of a polymer-associated reporter. Absolute oxygen measurement requires calibration of the complete formulation in its relevant environment.

Figure 5 outlines the mechanisms behind four optical readouts. A mechanical inference requires a force-coupled or otherwise physically calibrated response matched to the specified quantity. pH, oxygenation and localisation provide complementary microenvironment measurements. Table 2 therefore records each demonstrated setting and calibration.

### 5.1. Distribution and Activatable Fluorescence

Always-on fluorescence mainly reflects reporter exposure and retention. Tissue attenuation, imaging depth, acquisition settings, and photophysical state also contribute to the observed signal. The framework in Section 1.2 sets out the additional evidence needed to link a distribution image to mechanics.

An activatable signal has additional dependencies. Protease-sensitive fluorescence reflects substrate accessibility, enzyme activity, local probe concentration, and the fate of the cleavage product. A pH-responsive assembly reports a protonation-dependent transition in the environment reached by the polymer. Acidity and proteolysis may accompany matrix remodelling, but neither measures tissue stiffness directly; their association with mechanics must be tested in the model under study.

Hadzima and colleagues assembled cathepsin-cleavable fluorophore–quencher systems on RAFT-prepared pHPMA scaffolds [26]. Signal retention and tumour contrast changed depending on whether cleavage released the dye or the quencher. These related architectures allow a quantitative comparison in Table 2.

Ratiometric design need not require two emission colours. Yan and colleagues developed monochromatic ratiometric imaging nanoparticles in which Cy5 was quenched by Cy7.5 within ultra-pH-sensitive polymer micelles. Endosomal acidification dissociated internalised micelles and restored Cy5 fluorescence. Subsequent 808 nm irradiation photobleached the remaining quencher, revealing the total fluorescently labelled population. The pre-irradiation and post-irradiation images thus provided two operational states of the same emission channel. Controls representing the limits of zero and complete internalisation supported the separation of extracellular and intracellular exposure in tumour models. This approach avoids differential tissue attenuation between two detection wavelengths, but obtains its reference sequentially and through an imposed photochemical conversion [41].

The choice of reference should follow the biological question. For carrier uptake, the reference must represent material available for internalisation. For an enzyme-responsive probe, it should remain detectable after substrate cleavage. Zhang and colleagues conjugated a granzyme-B-cleavable, dye-labelled peptide to a semiconducting polymer; cleavage changed the dye-associated fluorescence and photoacoustic signals while leaving the polymer reference comparatively stable. That protease-dependent readout complements the transcript recognition used by SENSE, because the two approaches sample different stages of cytotoxic-cell function. Reading them together would test whether increased *Gzmb* transcription is accompanied by accessible enzyme activity, rather than assuming that transcription, secretion, and killing rise in parallel [27,57].

### 5.2. Distance-Dependent Quenching and Molecular Tension

Distance-sensitive reporters convert polymer extension or molecular separation into fluorescence. Förster resonance energy transfer between a donor and acceptor follows a different distance dependence from quenching by a metal nanoparticle, so the calibrations are not interchangeable. In the PEG–gold system, force inference combines the fluorophore–particle distance calibration, the polymer force–extension model, and the anchoring geometry [5].

Attachment to a biological receptor adds another layer of uncertainty. Ligand density can influence clustering, while the population of engaged tethers determines which forces contribute to an image. Polymer polydispersity, linker orientation, and nonspecific adhesion can broaden the response. The reported quantity must consequently be defined: a mean tension, a population-weighted signal or the fraction of activated reporters. An uncharacterised mixture cannot support a single universal force assignment.

Wang and colleagues paired receptor-level fluorescence from hydrogel-immobilised DNA tension probes with traction-force microscopy [25]. Fibroblasts altered the number of force-bearing integrins and their sampling of matrix rigidity, whereas T cells exhibited rigidity-dependent receptor forces. The mechanically defined polymer substrate provided controlled cell-level validation. This approach helps identify whether a fluorescence change reflects force per linkage or the population of engaged linkages. The study did not measure mechanics in animal tumours or relate the signal to treatment outcomes.

### 5.3. Calibrated Physical Measurements in Tumours

#### 5.3.1. Polymer-Assisted Local Microviscosity

Molecular rotors respond to the restriction of intramolecular motion through changes in non-radiative relaxation. A validated lifetime–viscosity calibration can convert this response into local microviscosity. Although lifetime depends less directly on fluorophore concentration than intensity, it still depends on the emitting species and the decay model. Aggregation, mixed compartments, or additional quenching pathways may invalidate that calibration.

The CT26 study by Shimolina and colleagues measured local microviscosity, providing a tumour-validated physical readout [6]. With separate lifetime–viscosity calibration, polymer-delivered BODIPY1 yielded a collagen-associated viscosity of 265 ± 16 cP, while non-polymeric water-soluble BODIPY2 yielded a tumour-cell value of 386 ± 19 cP. Because the formulations sampled different compartments, these values do not show that the polymer improved viscosity sensitivity, nor do they measure tissue stiffness. The work demonstrated imaging without establishing prediction of therapeutic benefit.

#### 5.3.2. Deformation-Based Solid-Stress Measurements in Tumours

Zhang and colleagues used fluorescent fibronectin-coated polyacrylamide inclusions to measure a complementary physical quantity in primary murine breast tumours and lung metastasis [24]. Fluorescence identifies the deformed sensor geometry, which a finite-element model converts into a stress field using characterised polymer elasticity separately. Mechanical sensing thus resides in the elastic polymer body; the chromophore does not itself respond to force. The cell-sized and tissue-sized inclusions had reported diameters of 28.7 ± 18.2 μm and 397 ± 69 μm, with corresponding elastic moduli of 0.21 ± 0.04 kPa and 0.38 ± 0.15 kPa (mean ± SD). Validation covered approximately 0.1–4 kPa for these calibrated inclusions. This range is not a universal tissue-stress detection limit.

This method measures deformation-derived solid stress at a larger spatial scale than a rotor. It requires known relaxed geometry, a mechanical model, sensor integration and optical access. The inclusion can also perturb its surroundings, while intravital-window and depth restrictions constrain longitudinal access. Although the study measured tumour mechanics, it did not establish a fluorescence-based predictor of treatment outcome. Its in-vivo mechanical inference is supported more directly than that of a synthetic network demonstration, but the method remains less suited to systemic molecular imaging.

**Figure 6:**
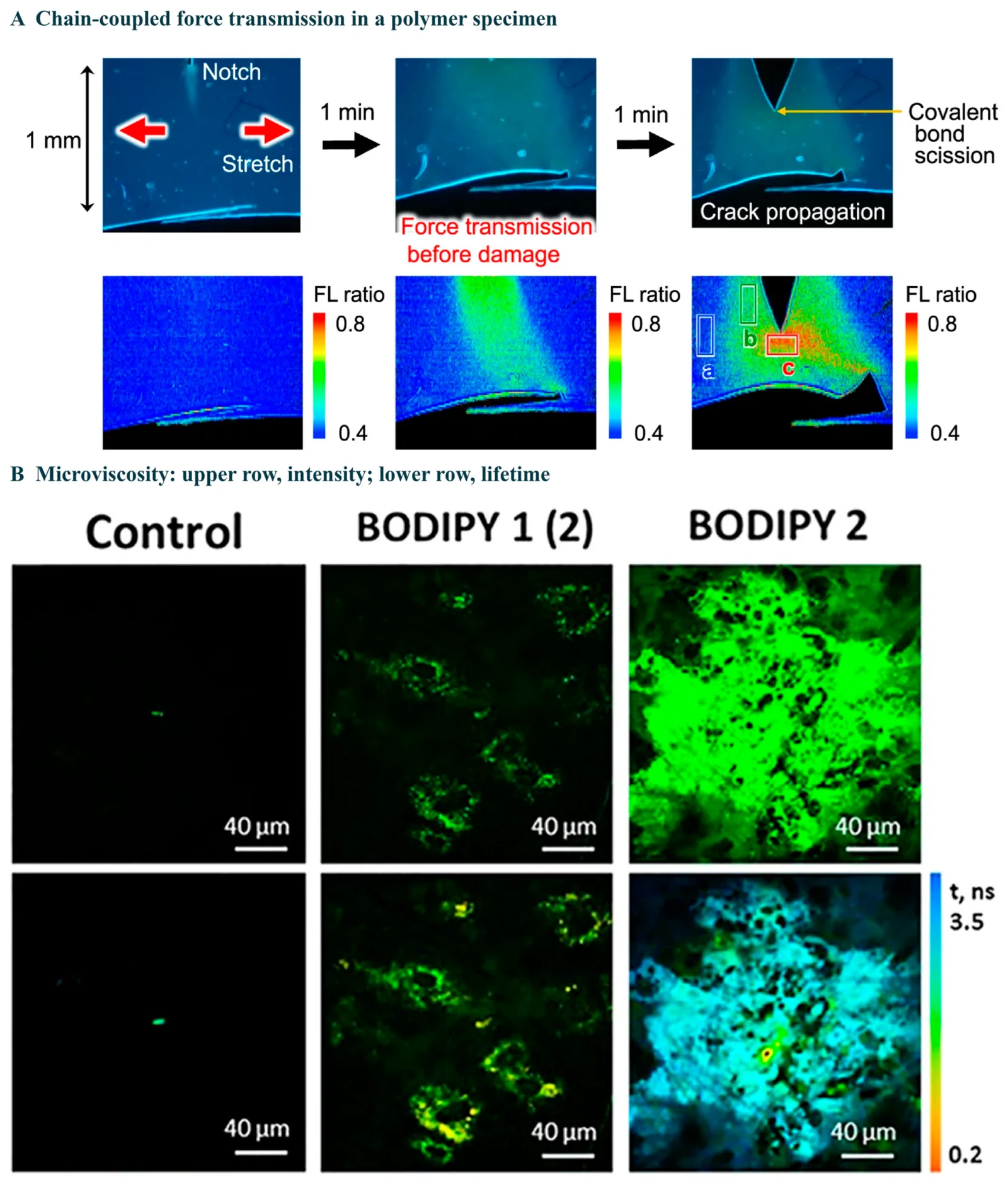
Materials-stage mechanical transduction and tumour-validated microviscosity imaging. (**A**) Tensile loading of a notched FLAP1-containing polycarbonate specimen, a synthetic-material demonstration without tumour validation. Excitation, 365 nm; strain rate, 2.0 × 10^−3^ s^−1^; ratio, FL525/FL470; length indicator, 1 mm [30]. (**B**) CT26 tumour imaging with BODIPY1 in polymeric brushes (40 min after intravenous injection; 3 mg/kg dye; brush dose disputed, Table 2) and water-soluble BODIPY2 (60 min; 3 mg/kg dye), alongside control tissue. The upper row shows two-photon fluorescence and the lower row shows lifetime. Excitation, 800 nm; detection, 409–680 nm; scale bars, 40 μm [6]. These formulations sample different compartments; numerical viscosity inference requires a formulation-valid decay and a separate lifetime–viscosity calibration. In the final ratio image of panel A, a marks an unstretched region, b a region behind the crack, and c the region ahead of the crack tip.

Figure 6 places a materials-only deformation experiment alongside calibrated tumour microviscosity imaging to compare their physical quantities and levels of evidence. The colour scales belong to different measurements and cannot support a common ranking of mechanical performance.

### 5.4. Oxygenation and Optical Contrast

Parshina and colleagues combined oxygen-sensitive PIr3 phosphorescence lifetime with endogenous NAD(P)H FLIM to image CT26 tumours [29]. Local injection and removal of the overlying skin provided access for microscopy. Cellular interactions altered probe decay, and the study did not establish intracellular oxygen calibration. The resulting oxygen-sensitive lifetime map supports complementary physiological monitoring but does not quantify absolute oxygen concentration.

NIR-II emission can aid imaging under specified tissue and detector conditions. Its spectral range alone does not make a signal mechanically specific: Hong demonstrated vascular imaging, and Tang demonstrated membrane-targeted photo-immunotherapy, with neither signal calibrated against tumour stiffness or membrane tension [4,40]. Table 2 compares their quantitative optical and formulation properties.

Tang and colleagues combined a BODIPY-containing fluorescent polymer with a separate cationic, cholesterol-bearing membrane-targeting polymer for NIR-II imaging-guided photo-immunotherapy. Reactive oxygen species (ROS)-responsive thioketal linkages in the fluorescent component enabled light-triggered degradation and release of the membrane-active component. The design links optical emission and polymer breakdown to membrane-directed injury. Its fluorescence and therapeutic endpoints support that application [40].

### 5.5. Afterglow and Excitation-Separated Imaging

Afterglow separates photon collection from the illumination that prepares the emitting state. Miao and colleagues demonstrated biodegradable semiconducting polymer nanoparticles with near-infrared afterglow and substantially higher imaging contrast than their fluorescence channel in mouse experiments. The persistent signal arises from stored chemical energy, so its decay describes a different process from the nanosecond excited-state decay measured by fluorescence lifetime microscopy. This distinction opens an additional design dimension: polymer composition can influence both the generation of the emitting intermediate and its subsequent consumption [58].

Jiang and colleagues used that chemistry to construct an acidity-responsive two-particle system. One population contained a photosensitiser in polystyrene-block-poly(acrylic acid) particles; the other carried an afterglow substrate and a pH-responsive surface. Acid-induced charge reversal brought the populations together, facilitating transfer of singlet oxygen and activation of the afterglow substrate. The resulting signal depended on an encounter between separately formulated particles, rather than cleavage of a quencher tethered to one polymer chain. Mouse tumour imaging established a biological application, while the matched pH-insensitive formulation connected activation to the intended assembly mechanism. The design therefore extends responsiveness from intraparticle structure to interparticle organisation [42].

Afterglow can also be initiated acoustically. Xu and colleagues developed sonoafterglow nanoparticles in which ultrasound-triggered singlet-oxygen generation activated an afterglow substrate. A peroxynitrite-responsive formulation coupled imaging to an imiquimod prodrug in mouse cancer immunotheranostics. This adjacent platform shows how changing the activation modality can address light delivery while retaining excitation-separated detection. It does not remove the need to characterise the local oxidant chemistry. Across these approaches, the measured signal reflects the initiating stimulus, reactive-species availability and the amount of activatable substrate, making illumination or ultrasound history part of the experimental measurement [59].

Chemiluminescent and afterglow polymers offer immune-associated chemical monitoring with reduced excitation background. Superoxide-responsive semiconducting-polymer reporters were compared with T-cell populations in tumour-bearing mice; self-immolative ROS reporters showed prolonged emission in inflammatory models [60,61]. Each provides biochemical information in its tested biological setting.

Ratiometric afterglow addresses a separate problem: both donor and acceptor intensities decrease after charging. Liu and colleagues assembled MEHPPV, a photosensitiser and an NO-responsive acceptor with Pluronic F127. Photo-oxidation generated an energy-storing polymer intermediate; subsequent energy transfer produced two afterglow channels whose ratio changed upon NO activation. The ratio remained more stable than either intensity during the tested decay and acquisition conditions. Macrophage experiments included an NO-scavenger control, and mouse studies linked the optical response to pharmacological modulation of tumour-associated macrophages. The recognition element supplies chemical selectivity, while the paired emissions compensate for shared signal decay. Excitation-free acquisition therefore improves contrast, but analyte recognition and quantitative referencing remain separate design requirements [62].

**Figure 7:**
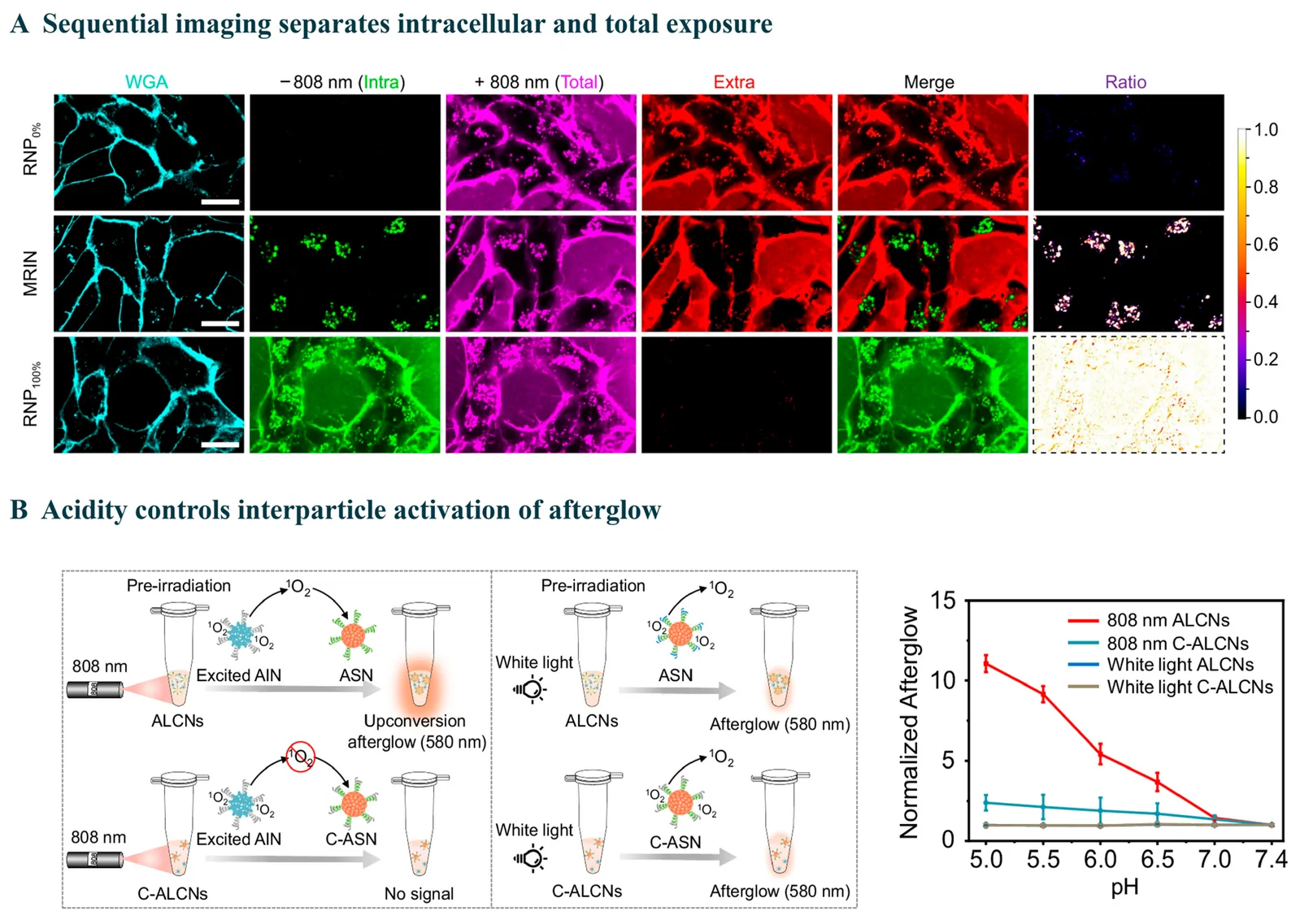
Activation schemes separate different sources of optical contrast. (**A**) Monochromatic ratiometric imaging of 4T1 cells after 0.5 h incubation at 37 °C with RNP0%, MRIN, or RNP100%. Cy5 images before and after 808-nm irradiation distinguish intracellular and total nanoparticle exposure; subtraction supplies the extracellular image. The ratio channel reports intracellular nanoparticle distribution, not cytosolic cargo delivery. Scale bars, 20 μm. (**B**) Afterglow-generation scheme and normalised pH-dependent responses of responsive ALCNs and pH-insensitive C-ALCNs after 1 min pre-irradiation with an 808-nm laser (1 W cm^−2^) or white light (0.1 W cm^−2^). Response measurements used 20 μg mL^−1^ ASN or C-ASN and 2 μg mL^−1^ AIN in HEPES buffer; values are mean ± SD, *n* = 3 independent experiments. AIN and ASN denote afterglow initiator and substrate nanoparticles, respectively. In panel A, minus and plus symbols indicate the absence and presence of 808-nm irradiation, respectively.

Figure 7 compares a sequential fluorescence reference for intracellular exposure with acidity-dependent afterglow activation. One separates operational exposure states; the other couples particle association to reactive-species transfer.

## 6. Polymer Fluorescent Probes in Treatment and Response Monitoring

**Figure 8:**
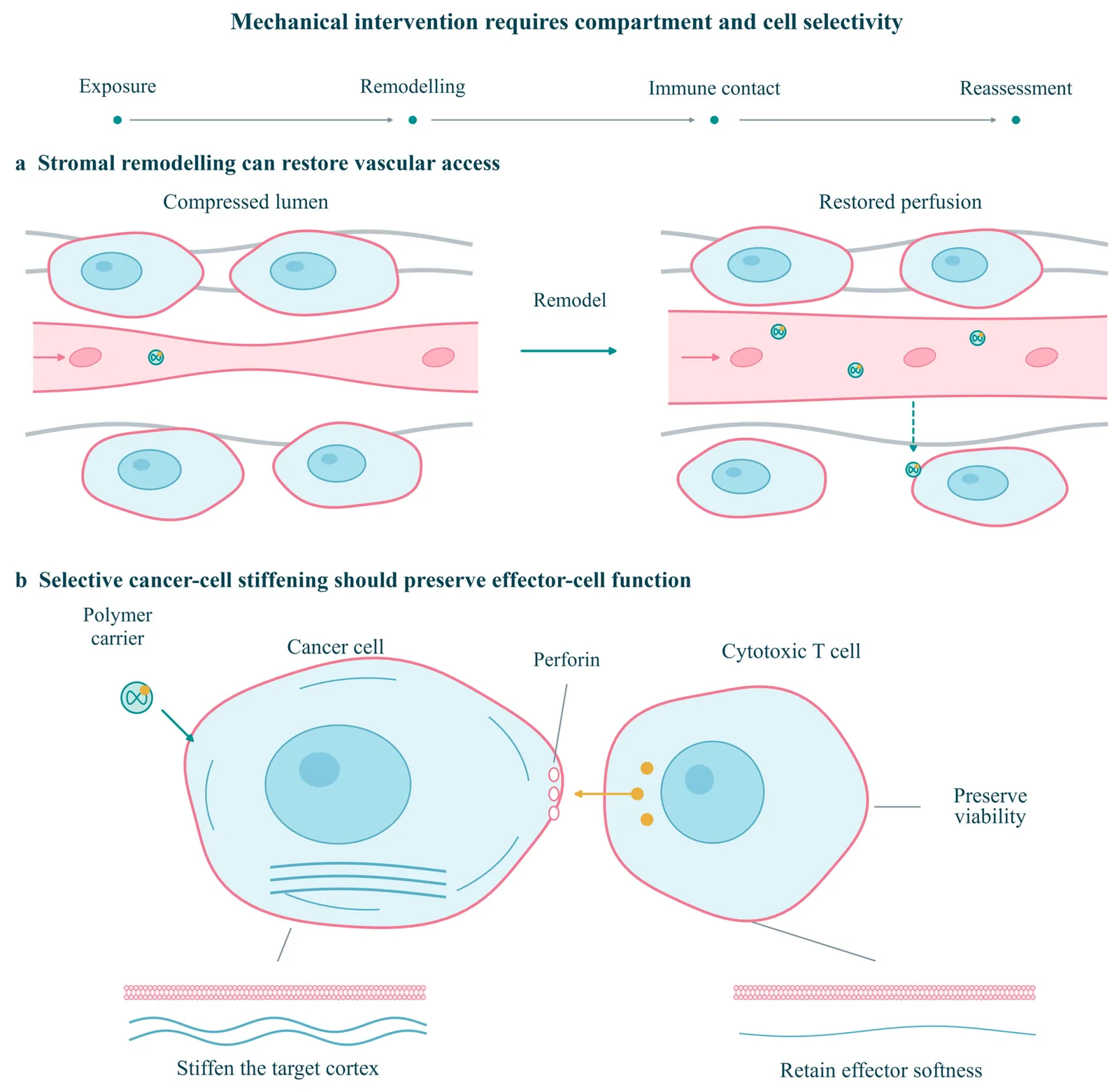
Mechanical intervention across tissue and cellular compartments. (**a**) Stromal remodelling can relieve vascular compression and improve perfusion. The dashed route indicates a possible increase in interstitial access, which still depends on endothelial transport and the formulation. (**b**) Cancer-cell stiffening and preservation of effector-cell softness are distinct design objectives. The membrane–cortex enlargements illustrate their different mechanical requirements; the perforin symbols depict delivery to the target-cell membrane. Cell-selective action must be verified because an intervention that stiffens cytotoxic lymphocytes can increase their own susceptibility to perforin. Orange dots associated with polymer carriers denote fluorescent labels; orange dots within the cytotoxic T cell denote perforin.

Figure 8 separates the timing of formulation exposure, early responses, and immune-cell function. This framework accommodates stromal decompression and target-cell stiffening as different, potentially coordinated interventions.

### 6.1. Stromal Remodelling and Therapeutic Access

Mechanically relevant therapy forms a separate category of evidence. Losartan relieved vascular compression and improved perfusion and drug delivery in preclinical breast and pancreatic tumour models [10]. Hong demonstrated a fluorescent conjugated-polymer vascular tracer in separate mouse experiments [4]. The reviewed literature does not contain a combined polymer-fluorescence test of losartan-induced decompression. Section 8.2 considers that proposed experiment.

Stromal intervention requires cell specificity because CAF populations have different functions. Broad depletion can remove tumour-restraining activity or increase immunosuppression [63,64,65].

The transport step under study must also match the route of administration. Bhalotia and colleagues used ultrasound-activated nanobubbles to alter collagen organisation and tissue stiffness, improving lipid-nanoparticle distribution and immune-cell transfection in mouse tumours. Both the nanobubbles and lipid nanoparticles were injected intratumorally. The work consequently addresses interstitial redistribution and cellular delivery after local deposition. It provides a useful experimental precedent for fluorescent polymer carriers: map spatial exposure, then measure the intended intracellular activity rather than inferring successful delivery from uptake alone [66].

Before analysing drug delivery, investigators assessed tranilast-associated matrix changes through tumour-opening experiments, mechanical tests, pressure measurements and perfusion histology [67]. Hyaluronan depletion and fibroblast-directed photothermal treatment provide further examples in which the mechanical intervention was independently assessed [68,69]. These studies support links between mechanics and treatment within their own formulations.

### 6.2. Cancer-Cell Mechanics and Immune Killing

Improved lymphocyte access does not guarantee successful killing. Collagen architecture influences T-cell movement and positioning, while events at the immune synapse depend on forces transmitted between an effector and its target. Basu and colleagues showed that cytotoxic T cells use mechanical force to potentiate perforin-mediated membrane damage. Lei and colleagues subsequently demonstrated that cholesterol depletion stiffened cancer cells and enhanced adoptive T-cell therapy in mice. These results locate a therapeutic opportunity at the target cell, distinct from softening the surrounding stroma to facilitate access [8,14,70,71].

Luo and colleagues combined IR783-labelled polyrotaxane with releasable methyl-β-cyclodextrin and optional diABZI loading [7]. They used independent assays of cholesterol modification and cortical mechanics, alongside therapeutic-outcome comparisons in tumour-bearing mice. IR783 tracked distribution without calibration to cortical modulus. The study connects a mechanically active polymer intervention with treatment efficacy, but does not establish its fluorescence readout as a predictor of efficacy. We retain the relative cell-mechanics measurements without converting them to an absolute modulus or an optical stiffness calibration.

**Figure 9:**
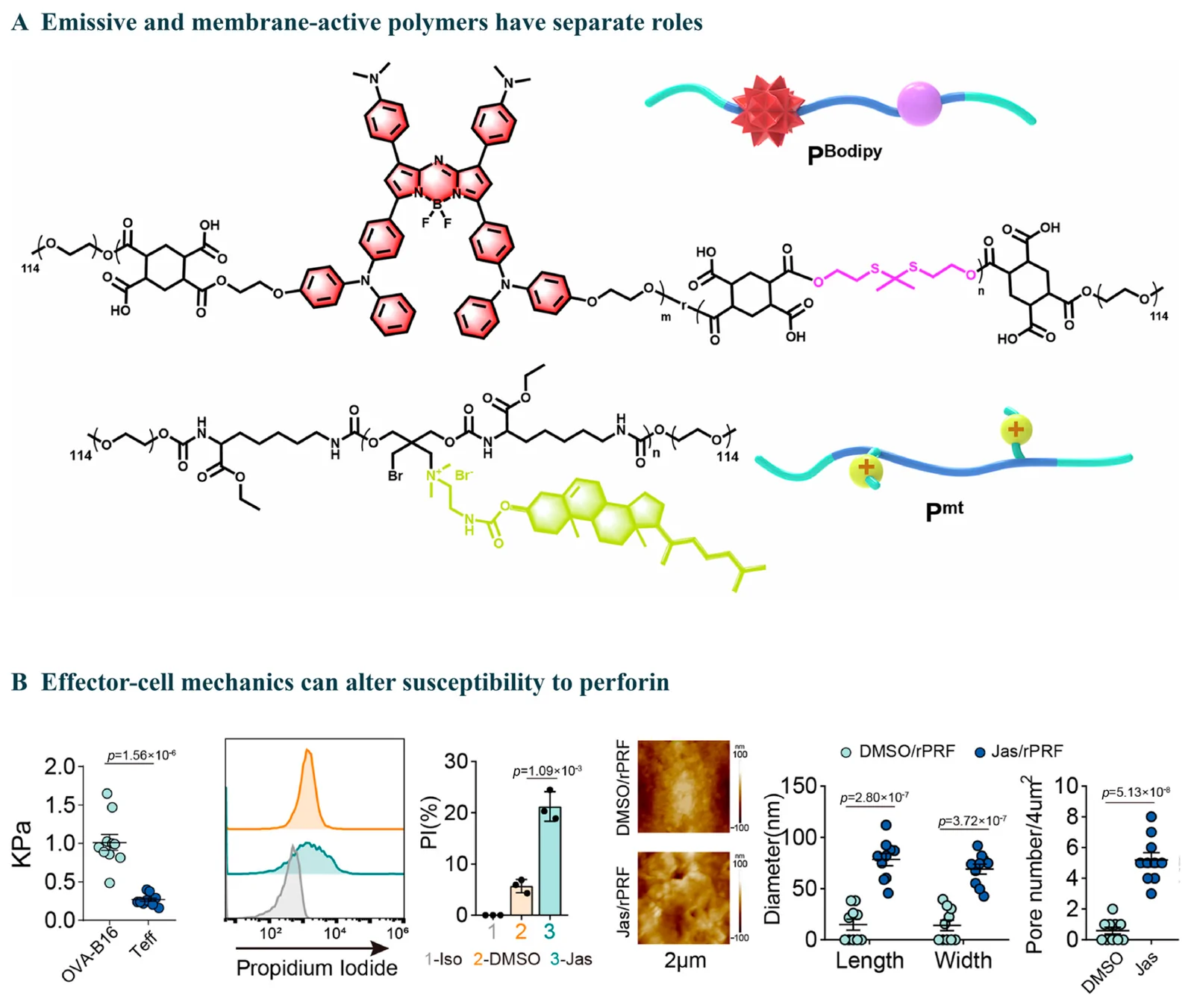
Membrane-directed polymer design and the need to preserve effector-cell function. (**A**) Structures of a BODIPY-containing fluorescent polymer with ROS-cleavable thioketal linkages and a separate cationic, cholesterol-bearing membrane-active polymer. Their distinct roles couple imaging and triggered degradation with membrane injury. (**B**) From left to right, the panels compare the stiffness of OVA-B16 cells and mouse effector T cells, show propidium-iodide positivity, and show AFM images and quantification of perforin-induced pores in human effector T cells pretreated with jasplakinolide (Jas; 200 nM, 12 h). Increased damage in the manipulated effector lymphocytes highlights a potential cost of nonselective stiffening. AFM scale bar, 2 μm; data are mean ± SD. PI analysis used *n* = 3 independent experiments. For the AFM comparisons, the source reports *n* = 10; each stiffness point averages at least 20 force curves from one cell, and pore measurements sample three randomly selected 2 × 2 μm regions per cell. Original *p* values are retained: the source specifies two-tailed Student’s t-tests for the stiffness comparison (left), one-way ANOVA with Bonferroni adjustment for the PI analysis (middle), and both procedures for the AFM pore measurements (right).

Cancer-cell and lymphocyte mechanics pose different therapeutic constraints. MRTF-associated actin remodelling increased cancer-cell susceptibility to cytotoxic lymphocytes, whereas softness protected effector cells from perforin-mediated injury [72,73]. These independent biological measurements support cell-resolved evaluation of membrane-active polymers. Figure 9 illustrates why a nonselective stiffness increase should not be assumed to be uniformly beneficial.

Figure 9 links the design of membrane-active fluorescent polymers with evidence that effector-cell mechanics can influence perforin susceptibility. Their juxtaposition highlights a selectivity requirement: delivery and physical effects must be resolved on both sides of the immune synapse.

### 6.3. Biochemical Readouts and Longitudinal Assessment

SENSE pairs a semiconducting-polymer reference with Gzmb-transcript-responsive DNA recognition [27]. Xu and colleagues followed treatment-associated changes in mice and used independent molecular and cellular assays. Their work links a biochemical optical readout to chemo-immunotherapy response. The readout concerns transcription, which is distinct from enzyme activity, synaptic delivery and killing, and is not a mechanical measurement. Table 2 states this limit alongside the measured optical target.

Neutrophil elastase provides another immune-associated target. Zhang and colleagues attached an elastase-cleavable, peptide-caged hemicyanine to a semiconducting-polymer backbone. Proteolysis activated the hemicyanine fluorescence and photoacoustic response, while the polymer channel served as a reference. Imaging in mouse cancer immunotherapy experiments connected the response with tumour-associated neutrophils. This architecture complements SENSE at a different biological level: one reads a transcript, and the other reads proteolytic activity. Neither measurement alone resolves every functional state of the corresponding immune population. Together with superoxide-responsive chemiluminescence and NO-responsive afterglow, these probes illustrate why response monitoring benefits from chemically distinct targets. Agreement among activity, cell abundance and killing would strengthen a mechanistic interpretation; disagreement can identify recruitment without effective tumour control [27,60,62,74].

An optical polymer can also organise the intervention whose consequences are later measured. Li and colleagues developed semiconducting-polymer immunomodulatory nanoparticles, SPINs, that generated singlet oxygen under ultrasound and released conjugated immunomodulators through cleavable linkers. The dual formulation combined the IDO inhibitor NLG919 with an anti-PD-L1 antibody. Release measurements and receptor-binding assays established activation, whereas constitutive near-infrared fluorescence tracked polymer distribution. In mice, the combined formulation supported sono-immunotherapy; the orthotopic pancreatic-tumour experiment in rabbits evaluated a sonodynamic formulation without the dual immune payload. Keeping these models distinct clarifies what acoustic activation adds: control over the timing and location of treatment, alongside an optical measure of exposure [75].

Enzyme-triggered polymer conjugates can also activate treatment. Cathepsin-B cleavage can initiate self-immolation and chemically generated reactive species; mouse immune-treatment outcomes were assessed separately [76]. The platform alters intracellular chemistry; its relevance here lies in distinguishing distribution, activation, and response.

In these biochemical monitoring studies, the recognition element specifies the target associated with fluorescence or afterglow. Calling a treatment-associated trajectory a mechanical biomarker requires measurement of a matched physical quantity and testing of its relation to outcome. Stronger immune-associated fluorescence alone does not support that general inference [27,62,75].

**Figure 10:**
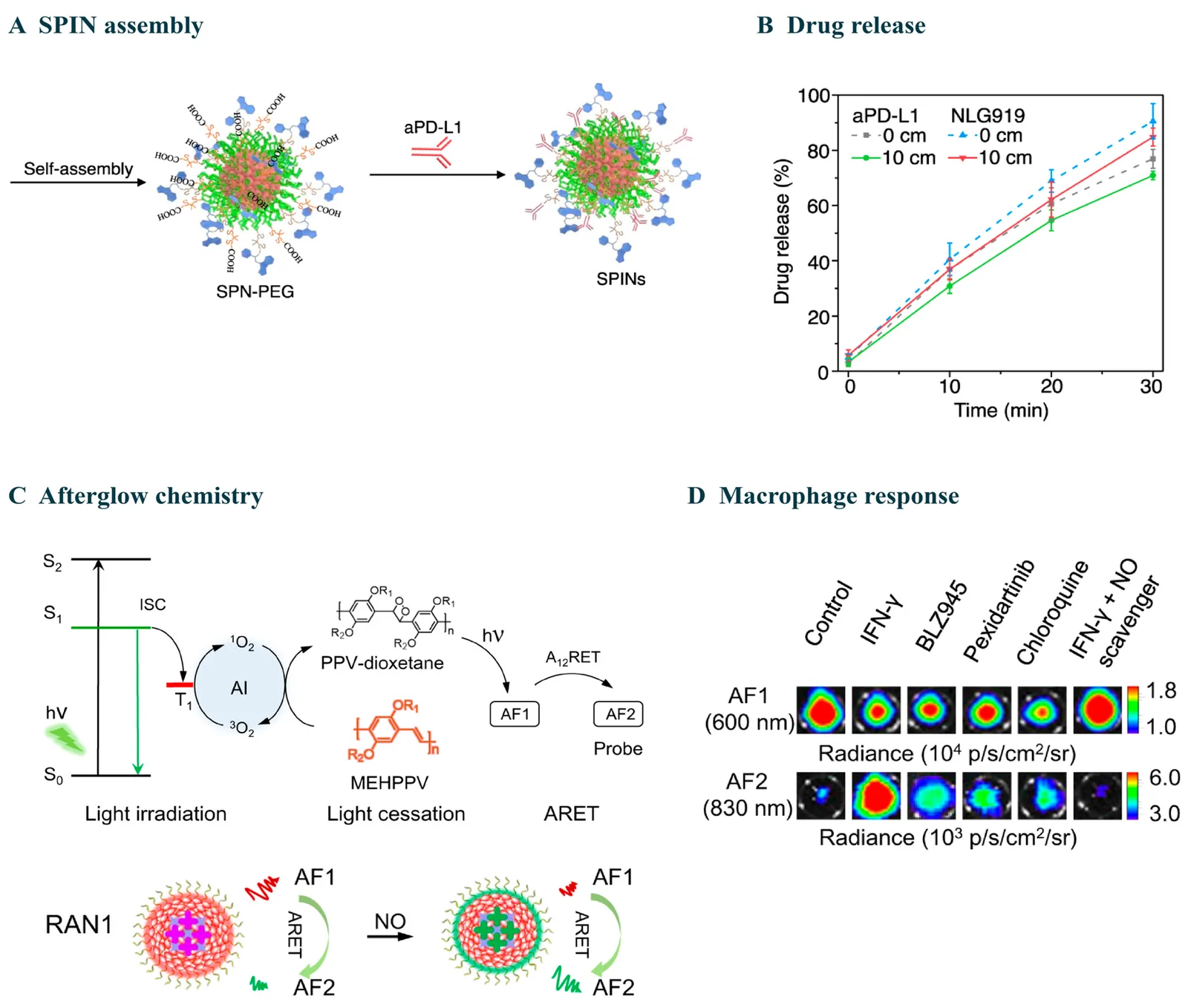
Polymer-triggered immune intervention and afterglow monitoring. (**A**) SPIN assembly and antibody functionalisation, selected from the right-hand component of original Figure 3a of Ref. [75]. (**B**) Anti-PD-L1 antibody and NLG919 release from SPIND2 through 0 or 10 cm of overlying ex vivo pork tissue (original Figure 3h of Ref. [75]): ultrasound, 1.0 MHz, 1.2 W cm^−2^, 50% duty cycle; formulation, 40 μg mL^−1^; mean ± SD, *n* = 4 [75]. (**C**) MEHPPV-based afterglow energy transfer and NO-responsive RAN1, from original Figure 2a and the top row of Figure 2b of Ref. [62]. (**D**) Representative RAW264.7 macrophage afterglow images following the indicated treatments, including an NO-scavenger control (original Figure 5b of Ref. [62]). RAN1 contained 10 μg mL^−1^ NRM and 25 μg mL^−1^ MEHPPV; 660-nm pre-illumination at 0.80 W cm^−2^ for 30 s preceded 30-s acquisition without excitation. AF1 and AF2 denote the two emission channels; radiance scales are retained. The authors report three experimental repeats [62].

Figure 10 compares a polymer platform that releases immunomodulators after ultrasound exposure with a separate polymer afterglow system that follows an immune-associated chemical signal. Reading intervention and response together requires independent tests of release, biochemical specificity, and cell function.

## 7. Experimental Design and Quantitative Validation

### 7.1. Match the Model to the Delivery Step

An experimental model is useful when it retains the interface that the probe is intended to cross or measure. Hydrogels can separate initial stiffness from stress relaxation and ligand density [9]. Spheroids reveal interstitial penetration and local cell responses, but lack a perfused vascular barrier [55]. Endothelial models address transport across vessels, while immune co-cultures retain the contact and killing events needed to test a synaptic mechanism [51,71]. Comparisons across these systems are strongest when they share a defined perturbation and compatible measurement times.

Synthetic networks can make such comparisons more discriminating. Peng and colleagues developed DNA-crosslinked polymer matrices in which sequence design tuned stress-relaxation times across four orders of magnitude, with rheological validation and demonstrations of three-dimensional cell and organoid culture. The platform provides a materials-based means of varying mechanical timescales; the reported cultures themselves do not establish a tumour-delivery model [77]. For probe validation, an appropriate extension would hold adhesive-ligand density and initial modulus approximately constant while varying relaxation, then measure both transport and reporter response. The network’s permeability and potential affinity for the probe must be characterised independently, because altered crosslinking may change molecular access as well as mechanics. A formulation that produces the same calibrated response in acellular networks but different signals in cell-containing matrices would help separate material-level interference from biological remodelling.

Biological and material properties need to be characterised together. For a deformable carrier, a change in crosslinking may also alter swelling, size and reporter loading. For a matrix-modifying formulation, collagen organisation, vascular function and recipient-cell identity establish where the intervention acts. Spatial molecular measurements can identify the responding cell state; an independent force or deformation assay establishes the accompanying physical change [3,24].

### 7.2. Formulation Integrity and Calibration Controls

The framework in Section 1.2 concerns the complete formulation used in the biological experiment. Table 2 records its reported size, molecular weight, chromophore content, loading terminology, stability conditions, and administration. Each stability test addresses a particular property: a stable DLS diameter cannot establish chemical integrity or cargo retention, and photostability cannot establish colloidal stability. When cleavage or disassembly contributes to the mechanism, the formulation and any released emitting species should be characterised separately.

The assigned polymer role guides the choice of controls. For an M-role sensor, loading geometry and mechanical calibration must be known. A C-role carrier requires reporter-retention and localisation controls; a G-role assembly requires an independent measure of activation; an E-role emitter requires matched photophysical characterisation. Non-load-bearing or non-responsive analogues distinguish these interpretations more directly than comparisons of brightness [5,6,43].

Temperature and protonation jointly govern the transition of digital fluorescent copolymers [43]. Their live-cell switching illustrates how to control for such interference.

### 7.3. Connect Exposure with a Physical Intermediate and a Functional Outcome

A mechanistic experiment follows the sequence proposed for the treatment. After stromal remodelling, the relevant steps may be renewed perfusion, increased tumour-cell exposure, and functional cargo delivery. For a membrane-active polymer, they may be cholesterol modification, a change in cortical mechanics and increased immune killing. Early measurements before extensive tissue destruction help establish whether the physical response precedes regression.

Across these studies, the strongest comparisons isolate one link in the proposed sequence. Corona manipulation tests a contribution to circulation; genetic YAP perturbation tests a recipient-cell mechanism; paired rheology and transport measurements test a matrix-level consequence. Each remains specific to the material, compartment, and model used [48,56,67]. Cross-study comparisons must account for dose, acquisition time, and sample definition before differences in apparent signal are interpreted biologically. Similar-looking images from different laboratories cannot establish relative efficacy.

Controls should address a plausible competing explanation. Empty carriers help assess material effects, while matched non-responsive analogues test contributions from activation and distribution. Rescue experiments can test dependence on the proposed pathway or physical variable. Illumination belongs in the same analysis because heating, bleaching, and photodynamic injury can alter the tissue during measurement. Table 3 relates common optical observations to experiments that can resolve their interpretation.

### 7.4. Preserve Spatial and Statistical Structure

Repeated imaging can connect the duration of a physical response with the interval of therapeutic exposure. A pretreatment baseline, an early measurement, and later functional endpoints provide more information than an isolated endpoint after regression. Recovery and repeat dosing matter when a membrane or matrix response is transient. Imaging settings and processing must remain comparable over the sequence.

Pixels and cells sampled from one animal do not represent independent animals. Analysis should retain the nesting of observations within samples and account for repeated measurements. Predefined compartments and spatial distributions can expose responses concealed by a whole-tumour average. Exclusions require reasons independent of the desired result, and a candidate response biomarker needs evaluation in an independent dataset. These choices determine whether an apparent association can support a treatment decision.

## 8. Enabling Technologies and Future Applications

### 8.1. Materials-Stage Platforms Without Tumour Validation

Kotani and colleagues used a flapping fluorophore to demonstrate force-dependent colour changes in synthetic polymer specimens [30]. Muramatsu and colleagues later observed reversible fluorescence during tensile loading of rotaxane-containing polyurethane–urea hydrogels [32]. Mechanical coupling is essential to both transduction mechanisms, but neither study validated sensing inside a tumour; Table 2 and Figure 6 identify their evidence level accordingly.

Hydrogel studies extend mechanical transduction into aqueous networks. A water-soluble loop-mechanophore additive responded reversibly to swelling, tension and compression, with optical changes accompanying stress relaxation [31]. Rotaxane hydrogels coupled mechanical shuttling to dequenching and energy transfer [32]. In both, swelling and network chemistry can confound the signal.

Phosphorescent Cu(I) crosslinks revealed heterogeneous loading in synthetic networks. In poly(*N*-acryloylsemicarbazide) hydrogels, processing-dependent hydrogen-bond association generated fluorescence [78,79]. Crosslinks couple mechanically to loading, whereas the hydrogel emission reflects material organisation without measuring current load.

Anthracene–maleimide linkages recorded hydrogel compression through irreversible chemical activation. Fluorescent-bead inclusions in tunable microspheres supported force inference from deformation models [80,81]. The persistent chemical record integrates loading history; interpreting geometric changes requires calibrated elasticity.

A roughly 200-μm polydiacetylene particle produced optical changes in a controlled vessel-mimicking microchannel [82]. Its nonlinear response depended on flow, viscosity, suspended objects, and solution chemistry. The tested size and geometry support a microfluidic proof of concept, with no demonstration of an injectable tumour nanoprobe.

**Figure 11:**
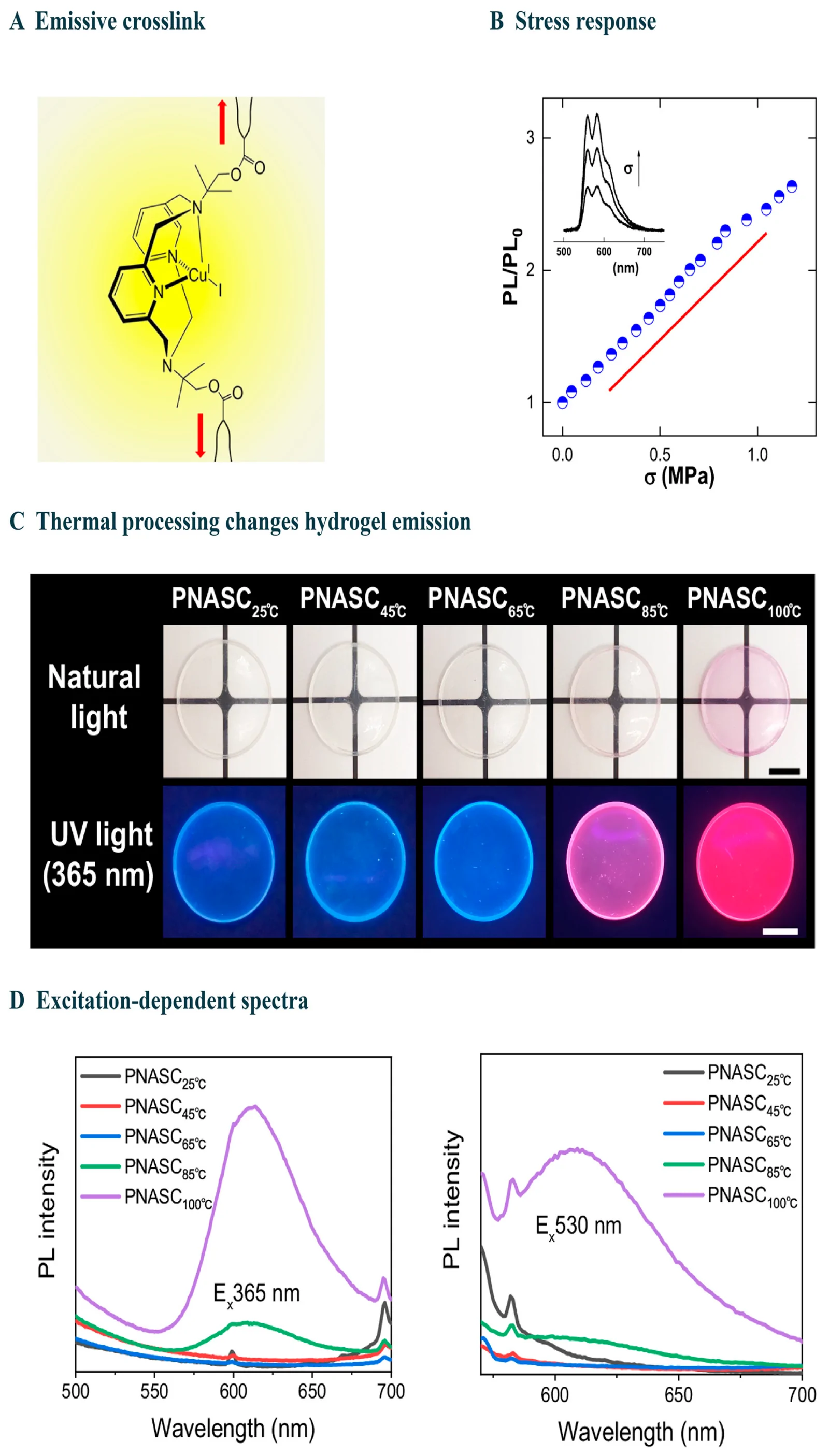
Materials-stage comparisons without tumour validation. (**A**,**B**) Cu(I)-containing emissive crosslinks and normalised photoluminescence plotted against tensile stress show mechanical coupling in a synthetic polymer network. (**C**,**D**) Thermally processed poly(*N*-acryloylsemicarbazide) hydrogels and their spectra show processing-dependent fluorescence. Temperature labels specify preparation conditions and do not provide a physiological sensing calibration. Scale bars in C, 1 cm; excitation in D, 365 nm (left) and 530 nm (right). A,B reproduce original Figure 1a and Figure 3a of Zheng et al. [78]; C,D reproduce original Figure 2a,e,f of Wu et al. [79]. Red arrows in panel A indicate opposing tensile loading directions.

Figure 11 compares force-coupled phosphorescence with intrinsic emission governed by hydrogel processing. Both illustrate how a polymer network can generate an optical response, while the perturbation and calibration determine what that response means.

### 8.2. Proposed Experiments and Treatment Timing

Reference channels can distinguish changes in probe exposure from activation if their distribution and optical attenuation are characterised. Pan and colleagues used co-injected activatable and always-on nanoparticle modules to examine fluorescence amplification independently of some accumulation effects. Their results also showed that changing core hydrophobicity altered biological interactions. A reference therefore works within a validated formulation and acquisition scheme, rather than supplying a universal correction [28].

The following combinations are proposals for future experiments; the cited studies did not test them. NIR-II tracer imaging before and after stromal decompression could resolve perfusion timing, provided that independent stress measurements and matched exposure controls were acquired. A calibrated rotor could test whether treatment changes local microviscosity within a fixed compartment. Separate indentation or rheology would still be needed to assess stiffness or relaxation. Pairing a physically calibrated tension reporter with a biochemical immune reporter could test whether receptor loading changes before treatment response [4,6,10,27].

Polymer photosensitiser chemistry also offers ways to improve therapy. Glycol side chains supported water-associated radical generation, and a Type-I photosensitiser–niclosamide conjugate supported hypoxia-tolerant photo-immunotherapy [44,83]. These are enabling treatment technologies without a demonstrated mechanical sensing function.

### 8.3. Clinical Evidence and Remaining Gaps

In the HALO 109-301 trial, adding pegvorhyaluronidase alfa to nab-paclitaxel and gemcitabine did not improve overall survival in hyaluronan-high metastatic pancreatic cancer [84]. The result limits claims that a plausible mechanism for stromal delivery guarantees clinical benefit. None of the fluorescent polymer measurements in Table 2 has been prospectively validated for patient selection or treatment decisions. Preclinical calibration, delivery gain, treatment association, and clinical utility each require their own endpoint.

### 8.4. Reproducible Manufacture and Practical Imaging

The structural features that govern polymer transport may also control its signal. Molecular-weight distribution, dye loading, free fluorophore, particle size, and activation threshold can vary between batches. Release specifications should therefore include a functional optical measurement under defined reference conditions alongside chemical identity and purity. When deformability or disassembly is central to the application, it belongs among those measured properties [3,85].

Practical access and clearance constrain even the strongest physical measurements. Studies using an implanted intravital window, exposed tumour microscopy, or local sensor placement have different requirements from non-invasive systemic imaging. Persistent material and released dyes may also follow different clearance routes. Table 2 specifies the route and experimental setting, allowing comparison without the assumption of a shared clinical workflow.

Selection should follow the measurement task. PEG tethers suit receptor loading at an anchored cell interface; rotors suit local viscosity within a validated compartment; deformable fluorescent inclusions support spatial stress inference; and activatable carriers report the chemical target specified by their recognition element. Before claiming an advantage across platforms, photophysical brightness, uptake and therapeutic output should be compared within matched series [5,6,24,33,41,44].

## 9. Conclusions

Fluorescent polymer platforms contribute to tumour mechanobiology through distinct functions. Calibrated rotor lifetimes and deformable fluorescent inclusions have provided physical measurements in mouse tumours. PEG tension sensors and responsive networks provide measurements at the cellular or materials stage. Conjugated emitters and chemically activated carriers serve delivery, microenvironment imaging and therapy monitoring, each with a different evidential scope.

Among the mechanically active polymer systems compared here, the strongest evidence linking treatment to mechanics comes from independent assays, rather than a force-calibrated fluorescent signal. Biochemical response monitoring also cannot establish that an optical trajectory reports stiffness or predicts benefit. The quantitative tables preserve these limits and distinguish reported values, inapplicable metrics, and unverified reporting.

A useful next study would focus on one physical quantity and compartment, calibrate the measurement and establish formulation integrity under matched conditions. It would then test whether an early measurement changes a treatment decision or predicts a later functional outcome. The heterogeneous studies reviewed here support neither a universal normalisation nor a clinically validated polymer-fluorescence biomarker.

## Figures and Tables

**Table 1:** Framework linking polymer architecture to measurement and biological inference.

Polymer Design and Role	Optical Mechanism	Variable and Scale	Calibration and Interpretive Boundary
Anchored PEG spring (M) [5]	Distance-dependent gold quenching	Receptor tension, pN; cell–substrate interface	NSET distance plus force–extension geometry; cancer-cell culture, not tumour modulus
Brush carrying BODIPY (C) [6]	Rotor lifetime	Local microviscosity, cP; accessible tumour compartment	Species-valid lifetime calibration; aggregation/compartment control; not stiffness
Fluorescent elastic PA inclusion (M) [24]	Shape/fiducial tracking	Solid stress, kPa; cell/tissue scales	Relaxed geometry, measured bead elasticity and mechanical model; intravital access required
Hydrogel-anchored tension probes (M) [25]	Thresholded force-induced unquenching	Molecular loading, pN; controlled culture interface	Threshold/temperature and ligand geometry; substrate kPa is distinct from receptor pN
Conjugated backbone (E), dispersing shell (C) [4]	Constitutive NIR fluorescence	Vascular exposure or reporter distribution	Photophysics, dose and acquisition; independent stress assay required for decompression
IR783-polyrotaxane (G/C) [7]	Distribution label on active carrier	Independent cell-mechanics intervention and therapy	AFM and functional outcomes are separate from fluorescence; no optical stiffness transfer function
Chemical gate or reference assembly (G/C/E) [26,27,28,29]	Protease, RNA, pH or oxygen-sensitive output	Specified biochemical/physiological target	Target-specific calibration; complementary monitoring, not a mechanical variable
Mechanophore polymer network (M) [30,31,32]	Force-coupled conformational/unquenching response	Applied material load/strain; synthetic network	Aqueous interference and mechanical coupling; no tumour validation

**Table 2:** Quantitative comparison of representative fluorescent polymer platforms. Part (**A**) compares demonstrated readouts, and Part (**B**) reports formulation and administration for the same platforms. The role labels are E = emission generation; M = essential mechanical coupling or calibrated deformable body; G = chemical gating; C = carriage/localisation. Reporting labels are NR = not reported in the examined accessible source; NV = not verified because relevant primary access or interpretation is incomplete; NA = not applicable. These labels do not imply that a field is absent from every supplement. Each value retains its specified formulation and assay conditions; no common performance ranking is inferred. Molecular weight/dispersity describe chains, particle PDI describes particles, and feed ratios do not establish retained loading. Other abbreviations: ΦF, fluorescence quantum yield; τ, excited-state lifetime; Đ, chain dispersity (Mw/Mn); PDI, particle polydispersity index; EE, encapsulation efficiency; ex/em, excitation/emission wavelength; i.v./i.t., intravenous/intratumoral; SI, supporting information; SP, semiconducting polymer; SNA, spherical nucleic acid; LOD, limit of detection; DOX, doxorubicin; GSH, glutathione. (**A**) Readout performance and demonstrated biological evidence. (**B**) Formulation properties and administration for the same platforms.

(**A**)				
**Platform and Role**	**Readout and Quantitative Operating Information**		**Demonstrated Setting and Outcome**	**Strength and Interpretive Limit**
Brush-delivered rotor [6] Role C	Green emission ~515 nm; two-photon excitation 800 nm. Tumour connective-tissue lifetime 2.24 ± 0.06 ns converts to 265 ± 16 cP using prior molecular-rotor calibration.		CT26 tumours in BALB/c mice; local microviscosity measured. No demonstrated treatment-outcome association.	Tumour physical measurement (local viscosity). Aggregation and compartment changes limit calibration.
Fluorescent PA inclusions [24] Role M	Validated stress span approximately 0.1–4 kPa. Bead elastic moduli 0.21 ± 0.04/0.38 ± 0.15 kPa for cell/tissue scales; deformation plus finite-element modelling, not intensity–force conversion.		Primary murine breast tumours and lung metastasis. Direct in-vivo solid-stress inference; no validated treatment-response predictor.	Tumour physical measurement (solid stress). Requires geometry, elasticity and FEM assumptions, plus intravital access.
PEG–gold tether [5] Role M	Excitation 488 nm (TIRF); ensemble 485/528 nm excitation/emission. DNA-ruler quenching distance d0 = 13.6 ± 0.3 nm; calculated response to ~25 pN; measured cell tension 1–15 pN, ~100 ms imaging.		HCC1143 breast-cancer cells on artificial substrate; no in vivo tumour mechanics or treatment outcome.	Controlled cell-level mechanics with a defined pN response. Requires anchored geometry and a force–extension model.
Hydrogel/DNA tension [25] Role M	Magnetic-tweezer-calibrated 17, 45 and 56 pN probes; 3 versus 30 kPa substrates. Threshold and sampling depend on temperature and linkage; not continuous universal force assignment.		Fibroblasts, T cells and cultured cancer-cell examples; no animal-tumour or anticancer outcome validation.	Controlled cell-level mechanics. Hydrogel compliance (kPa) and DNA force thresholds (pN) are distinct quantities.
IR783-polyrotaxane [7] Roles G/C	NIR IR783 fluorescence; specific excitation/emission settings NV. ΦF and excited-state τ NR in examined main/SI text. Fluorescence comparisons use matched IR783 concentration (S8); circulation half-life is not optical lifetime.		4T1 tumour study: MeβCD-mediated cell-mechanics modulation plus STING-agonist diABZI immunotherapy, with T-cell and survival outcomes. No calibrated IR783-based tumour-mechanics measurement.	Mechanically active therapy, independently assessed. Fluorescence follows distribution; the combined treatment cannot isolate mechanics alone.
pHPMA/cathepsin [26] Roles G/C	639/669 nm confocal excitation/detection. L-Q2:1F live-imaging tumour/background ratio 3.9 ± 0.9 at 24 h; 6QC comparator 2.3 ± 0.5 under that experiment.		4T1 mammary tumours; cathepsin imaging/contrast, not calibrated mechanics or treatment response.	Tumour protease imaging. Signal retention depends on whether cleavage releases the dye or the quencher.
iP5B5 HUNPs [28] Roles G/C	iP5B5 pH transition 5.22; ΔpHON/OFF ~0.20; >100-fold fluorescence activation. 48-h activation/reference ratio 2.88 ± 0.13 versus iP10 0.90 ± 0.23.		Multiple tumour models; fluorescence-guided mock resection detects <1 × 1 mm residual lesions, histologically checked. Chemical acidity/localization, not tumour-mechanics measurement.	Tumour acidity imaging with high activation contrast; a co-injected always-on module provides an exposure reference.
SENSE polymer–DNA [27] Roles E/G/C	Ratiometric Cy5 signal (ex 640 nm/em 660–680 nm) over an always-on SP reference (ex 710 nm/em 800–820 nm). Numerical LOD (3σ/slope), dynamic range and ΦF NV.		Subcutaneous 4T1 tumours in BALB/c mice after DOX chemotherapy (10 mg/kg i.p., three doses); in vivo ratiometric imaging compared with ex vivo CD8+ T-cell flow cytometry and tumour Gzmb qPCR (agreement metrics NV).	Tumour transcript monitoring with an internal SP reference. Gzmb mRNA level is not enzyme activity, completed killing or force.
mt-NPBodipy [40] Roles E/G/C	Nanoparticle absorption/emission maxima 792/914 nm, with NIR-II tail; 808 nm excitation. Nanoparticle QY NR.		CT26 mouse tumours; phototherapy/photoimmunotherapy, primary/distant tumour control and rechallenge. No calibrated membrane-tension measurement.	Imaging-guided photo-immunotherapy; light-triggered disassembly couples emission to membrane-directed injury.
MRIN/MRITN micelles [41] Roles G/C	pH transition 6.26, ΔpH 10–90% = 0.19, 110-fold Cy5 ON at 37 °C. Cy5 ex 630 nm, spectrum 645–750 nm/quantification 670 nm; Cy7.5 ex 780 nm/em 795–850 nm. 808-nm bleaching: 0.5 W/cm^2^, 4 min in vitro; 10 min in vivo. 24-h MRIN distribution: 24.2% intracellular/75.8% extracellular. ΦF/τ NR in examined main/SI text.		4T1 tumour compartment imaging and PDT comparison: combined extracellular + intracellular activation gives 94.5% tumour growth inhibition versus 52.2% intracellular PDT (source Figure 5 in Ref. [41]; 3-h drug-light interval). Compartment fraction is not cytosolic drug-release efficiency.	Separates extracellular and intracellular exposure in one emission channel. The reference is obtained sequentially, and imaging and therapy analogues differ.
PIr3 [29] Roles E/C	PIr3 em 605 nm. Water ΦP (degassed/aerated) = 8.79/5.77% (ex 360 nm); τ (ex 375 nm): 2.04 ± 0.03/1.41 ± 0.08 μs. PBS/BSA/DMEM τ = 1.31 ± 0.05/1.41 ± 0.05/1.33 ± 0.09 μs. QY Stern–Volmer Ksv = 0.00324 mmHg^−1^. In vivo two-photon ex 750 nm/em 570–640 nm; NAD(P)H em450–490 nm simultaneously. Absolute intracellular pO2 calibration was not performed.		CT26 tumours in BALB/c mice: oxygenation/metabolic spatial imaging; no tested treatment response. Intravenous delivery gave insufficient tumour accumulation (Table 2).	Simultaneous oxygen-sensitive PLIM and NAD(P)H FLIM. Absolute intracellular oxygen calibration absent; local injection required.
ASN/AIN afterglow [42] Roles E/G/C	Fluorescence ex 480 nm/em 590 and 793 nm; IVIS ex 480/em 580 nm. Afterglow precharge 808 nm, 1 W/cm^2^, 1 min; acquisition without illumination. pH7.4 → 5.0 gives 11.3× activation; afterglow half-life ~2 min under the reported assay, variable with power/dose/precharge. ΦF/excited-state τ NR in examined text; afterglow half-life is not τ.		4T1 subcutaneous tumours, HepG2 orthotopic liver tumours, and 4T1 pulmonary metastases imaged; biotin variant also tested in B16F10 xenografts. No therapeutic intervention tested. Tumour-acidity contrast is not tissue mechanics.	Excitation-free acidity imaging with a matched pH-insensitive control. Afterglow decay depends on precharge conditions.
Digital pH polymers [43] Role G	1 at 50 °C: ex 450 nm, pH 7 → 11 gives em 605 → 574 nm, ΦF 0.046 → 0.20, τ 3.3 → 9.8 ns; intensity a = 3.69 ± 0.34, apparent pKa = 8.68 ± 0.08, fluorescence output changes from 90.9% to 9.09% over 0.52 pH units. 7 at 37 °C: pKa = 8.58 ± 0.02, a = 2.78 ± 0.39. Cell-9 FLIM ex 470 nm/em 500–700 nm; τ 3.8 ± 0.16 ns (pH 5.5/22 °C) versus 7.4 ± 0.12 ns (pH 8.0/37 °C), *n* = 3–8 cells.		Live HeLa-cell logic operation under imposed pH/temperature; no tumour-bearing animal imaging or treatment test.	Intensity and lifetime switching enable pH/temperature logic. Temperature must be controlled when pH is inferred.
pDA/PEG NIR-II [4] Roles E/C	Absorption 654 nm; emission 1047 nm; 808 nm excitation. Quantum yield ~1.7%, relative to IR-26 assumed 0.5%, integrating 900–1500 nm. Vascular imaging 25.6 frames/s.		Targeted cancer-cell staining; in vivo vascular imaging principally healthy mice; no calibrated tumour-mechanics or therapy endpoint.	Fast NIR-II vascular imaging. Quantum yield is relative to an assumed IR-26 reference.
FLAP networks [30] Role M	Excitation 365 nm; PU force ratio I525/I474. DFT predicts ~100 pN planarization threshold, not a measured single-chain calibration. Unstretched rubbery PU1 (10% TEA): ΦF 0.28, τ 6.9 ns at 470 nm. Unstretched PC1: ΦF 0.30, τ 6.8 ns at 470 nm (SI).		No tumour/cell testing or treatment outcome; nanoscale force redistribution in synthetic polymer networks.	Ratiometric readout of nanoscale force redistribution. The theoretical pN threshold is not an animal-tumour detection limit.
Loop-additive hydrogel [31] Role M	Hydrogel ex 405 nm, em 478 nm; photographs use 395 nm. Stretch 0 → 430% gives ~6-fold emission increase (20 → 120 CPS); swelling ~700% relative mass increase gives 4.8-fold. Solution spectra normalized to BPEA reference at 478 nm; normalized intensity is not ΦF. Absolute ΦF/τ NV; molecular force calibration NR in examined main text.		No cells, tumour model or treatment endpoint; material proof of concept.	Reversible readout of hydrogel strain and stress relaxation. Swelling interferes with the signal.
RotAnBP hydrogel [32] Role M	Hydrogel ex 490 nm/em 660 nm, ΦF 0.02 unloaded (dry film 0.05). Hydrogel Young’s modulus 1.19 ± 0.04 MPa versus dry 29.5 ± 7.8 MPa; extensibility ~1200%; force-activated signal up to 7-fold. Fluorescence recovers gradually after unloading. τ NV (SI). Absolute per-chain force calibration NR in examined main text.		No tumour/cell/treatment study; synthetic hydrogel force reporting.	Force-controlled FRET in a highly extensible hydrogel, with gradual recovery after unloading. MPa bulk modulus is not molecular force or tumour modulus.
(**B**)				
**Platform**	**Size and Polymer Molecular Weight**	**Probe Content and Loading Terminology**	**Stability Endpoint and Conditions**	**Administration**
Brush-delivered rotor [6]	Carrier 100–150 nm quoted from precursor characterization; method not restated for the injected preparation. Mn/Mw/Đ NV.	1:1 dye:brush-repeat-unit molar feed; 24 h mixing disaggregates dye. Not measured loading efficiency; retained loading/EE NV.	Aggregation observed in some compartments; formal serum-challenge duration NV. Biexponential aggregated decays cannot yield valid viscosity.	Tail-vein: 3 mg/kg BODIPY1; SI S4 labels (1)/(2) use 8/14.8 mg/kg brush. The 2.24 ns result is label (2); the Figure 5 caption of Ref. [6] instead gives 8 mg/kg. Separate biodistribution: 5 mg/kg dye plus 12 mg/kg brush.
Fluorescent PA inclusions [24]	Cell/tissue-scale diameters 28.7 ± 18.2/397 ± 69 μm (mean ± SD); not nanoparticles. NA finite Mn/Mw for crosslinked PA networks; calibrated elastic modulus, not chain mass, defines stress inference.	Covalently attached fluorophore and fibronectin surface coating; quantitative retained fluorophore content NV; encapsulation efficiency NA to mechanical inference.	Longitudinal imaging limited by intravital-window access (less than about 2 weeks); chemical/cargo stability NR in examined text.	Tissue-scale placement during tumour induction; cell-scale beads co-injected or delivered intravenously for lung-arrest experiments; mass-normalised systemic probe dose NA to the local stress calibration.
PEG–gold tether [5]	15 nm gold core (TEM/supplier); hydrodynamic size NR. PEG nominal 3400 Da; MALDI 80 ± 7 repeat units; Mn/Mw/Đ NR.	Binary functionalization permits 1–10 reporters/particle; characterized 8.2 ± 0.3 Alexa488-PEG82-SH/particle. Condition-specific covalent surface density; encapsulation EE NA.	Reversible cell-force response; quantitative serum stability duration NR.	NA systemic administration; substrate-bound assay. Surface preparation: 2 nM particles, 10 min.
Hydrogel/DNA tension [25]	NA particle diameter: immobilised hydrogel culture substrate. NA finite network Mn; PA network formulation-specific chain metrics NV.	Ligand/probe surface density NV; encapsulation efficiency NA.	Systemic serum stability NA; culture-assay long-term reporter integrity NV.	Immobilised culture assay; systemic dose NA.
IR783-polyrotaxane [7]	DLS, mean ± SD (*n* = 3): RDPN 94.09 ± 6.8 nm; NPN 131.71 ± 9.4 nm; RDPN@diABZI 142.7 ± 8.5 nm. TEM shows spheres; numerical TEM mean NR in examined main/SI text. RDPN polymer Mn 39.4 kDa by GPC; PEG-PDS axle Mn 16.8 kDa. Final-polymer Mw/Đ NV (no verified numerical figure readout).	Two IR783 endcaps per proposed architecture; measured IR783 wt% NR in examined sources. diABZI loading/encapsulation efficiencies NV because reported definitions and feed conflict.	DLS size followed for 14 days in PBS, RPMI-1640 + 10% FBS and RPMI-1640 + 2% mouse serum (Figure 2f and Supplementary Figures S9–S11 of Ref. [7]). This assesses size stability, not chemical dye retention.	i.v. tumour distribution/treatment and additional i.t. experiments; numerical administered formulation/IR783 dose NV. Blood IR783 t1/2: RDPN 1.40 h versus free IR783 0.31 h; diABZI t1/2: loaded 1.5 h versus free 0.25 h.
pHPMA/cathepsin [26]	Soluble polymer conjugate; hydrodynamic diameter NV. P3 precursor: Mn 54/Mw 65 kg/mol, Đ 1.20 (SEC); L-Q2:1F calculated Mw′ 75 kg/mol. Final-conjugate Mn/Đ NV.	Approximately 2 fluorophores/chain across designs. L-Q2:1F: F 6.1 wt% (2.4/chain), Q 6.2 wt% (4.7/chain), Q:F 1.9. Isolated conjugate 8.6 mg; percentage coupling yield NV; encapsulation EE NA.	Quantified formulation/serum stability duration NV; tumour retention does not establish chemical stability.	Intravenous 6.25 nmol reported probe in 100 μL PBS with 10% DMSO; molecular counting basis NV.
iP5B5 HUNPs [28]	DLS ~30 nm at pH 7.4; ~8 nm at pH 4.8 (1 mg/mL, room temperature). Nominal PEG 5 kDa; whole-polymer Mn/Mw/Đ and particle PDI NR in examined main/SI/source data.	Turn-ON iP5B5-ICG: 1.19 ICG labels/chain, 49.6% covalent conjugation efficiency (UV spectrophotometry; dye:AMA feed 2.40:3). Encapsulation EE NA. The separate always-ON construct averages ~0.5 dye/chain.	Fresh mouse plasma, 37 °C, 24 h: preserved OFF-state fluorescence quenching; not a DLS-stability measurement.	i.v. 20 mg/kg turn-ON HUNPs; dual cocktail adds 40 mg/kg always-ON reference.
SENSE polymer–DNA [27]	DLS/zeta and TEM measured; numerical SENSE diameter NV. SP-N3 backbone Mw 24.1 kDa (SI); Mn/Đ NR in SI. Maleimide-PEG1000 side chains anchor the DNA hybrids.	Feed 5 nmol SP-PEG-Mal per 0.4 nmol DNA hybrid (recognition:Cy5 signal strand 3:1 at annealing). Conjugation efficiency determined by urea release of Cy5 strands; numerical value NV. Encapsulation EE NA.	Cy5/SP fluorescence remained ~1-fold over 48 h at 37 °C in HEPES, saline, PBS, 10% serum–PBS and pH 5 MES; DNase I (0.5 mg/mL) plus GSH (2 mM) challenge; in 10 mM GSH, ~25% activation at 24 h versus ~95% for gold SNA (Supplementary Figure S7 of Ref. [27]). Tests false activation, not DLS size.	i.v. 50 μg SP per mouse into ~50 mm^3^ 4T1 tumours after DOX (10 mg/kg i.p., every other day ×3); IVIS imaging to 24 h. Blood half-life 14.7 h (Supplementary Figure S12 of Ref. [27]).
mt-NPBodipy [40]	mt-NPBodipy DLS ~92 nm; particle PDI 0.24; zeta −11 mV. Whole-polymer Mn/Mw/Đ NR in examined main/SI text.	PBodipy:Pmt 1:1 mass feed, not retained loading. Covalent chromophore mass fraction NR; encapsulation EE NA.	Formal serum/time stability NR. 808 nm, 10 min produces enlargement/disassembly: stimulus response.	i.v. 200 µL at 500 µg/mL; therapy concentration expressed as AzaTPABodipy equivalent.
MRIN/MRITN micelles [41]	MRIN DLS diameters labelled in S2c: 29.1 nm at pH 7.4; 30.5 nm after 808 nm irradiation at pH 7.4; 6.5 nm at pH 5.4. Numerical TEM mean and MRITN diameter NR in examined main/SI. PEG precursor 5 kDa; full PEG-b-P(DPA40-r-EPA40-r-AMA3) Mn/Mw/Đ NR in examined main/SI text.	SI recipe: MRIN UPS-Cy5_1:UPS-Cy7.5_3:blank UPS = 5:45:50%; subscripts indicate dyes/chain. Therapeutic MRITN UPS-Ce6_1:UPS-Cy7.5_3 = 50:50. These blend recipes are not dye-wt% or EE.	Fluorescence spectra monitored in fresh mouse plasma at 37 °C for 24 h, dye equivalent 0.25 μg/mL; PBS pH7.4/5.4 controls. Optical signal stability, not a quantified DLS/chemical integrity time course. MRITN blood elimination half-life 18.8 h (S15; Ce6-normalized fluorescence), separate from optical stability.	MRIN i.v. 75 μg Cy5/kg in bilateral 4T1 tumours; imaging 24 h. MRITN i.v. Ce6 0.75 mg/kg; PDT 660 nm, 400 mW/cm^2^, 10 min at 3/24 h; treatment schedules day 1/5 or 1/5/9.
PIr3 [29]	PIr3 DLS mean 26 nm in water at 0.1–0.2 g/L; TEM diameter NR in examined main/SI. PIr3 Mn 39.3 kDa, Mw 62.6 kDa, chain Đ 1.59 (THF GPC, polystyrene calibration; SI synthesis text).	Covalently incorporated Ir reporter; organic:Ir-bearing monomer feed/proposed repeat-unit ratio 40:1 (Scheme S4). Directly measured Ir wt% NR in examined main/SI; free-dye encapsulation EE NA.	CT26 intracellular phosphorescence lifetime ~1.15 µs maintained for 9 h (10 µM PIr3, DMEM + 10% FBS, 37 °C). Room-temperature lifetime endpoints in water, PBS, 5% BSA and DMEM + 10% FBS. Chemical/colloidal serum-stability duration NR in examined main/SI.	Successful tumour PLIM: intratumoral 2–4 mg/kg, 10–20 μL of 10 μM probe divided into 3–4 injections, 20–30 min before imaging. i.v. 20/40 mg/kg at 3/6 h gave insufficient tumour accumulation.
ASN/AIN afterglow [42]	DLS ASN 79.7 ± 2.3 nm, C-ASN 81.3 ± 1.9 nm, AIN 20.6 ± 1.6 nm. TEM shows spheres; numerical TEM mean NR in examined text. Supplier MEHPPV Mn 40–70 kDa; PS-b-PAA block Mn(PS) 2.7–3.3 kDa and Mn(PAA) 4–6 kDa, chain Đ ≤ 1.1. MEHPPV Mw/Đ NR in examined Materials.	Feed ASN: MEHPPV 0.25 mg/C18-PEG-DA 2 mg; AIN: NCBS 0.05 mg/PS-b-PAA 0.4 mg. These are feeds, not retained loading/EE. Final ‘ASN/AIN concentration’ is normalized to MEHPPV/NCBS mass, respectively; assays commonly 20:2 μg/mL.	Stored dark at ~4 °C, duration NR in SI preparation. pH-dependent charge monitored to 6 h is activation, not colloidal stability. Blood half-lives 11.9/33.6 min (ASN/AIN; i.v. 8/0.8 mg/kg) differ from afterglow decay.	4T1 local imaging i.t./i.m. ASN 1/AIN 0.1 mg/kg; orthotopic liver and metastatic imaging i.v. 4/0.4 mg/kg (MEHPPV/NCBS basis).
Digital pH polymers [43]	Particle DLS/TEM NA to the principal soluble-chain probe; numerical globule/aggregate dimensions NV. Table 1, PS-calibrated GPC: copolymer 1 Mn 40.0 kDa, Mw 120 kDa, Đ 3.01; 7 Mn 47.2 kDa, Mw 108 kDa, Đ 2.29; cell-logic 9 Mn 55.5 kDa, Mw 118 kDa, Đ 2.12.	Measured unit ratios (thermoresponsive:proton-binding:DBD-AA): 1 = 87.3:12.7:0.038; 7 = 90.7:9.3:0.054; 9 = 91.2:8.8:0.77. These are copolymer compositions, not encapsulation EE (NA).	After cellular incorporation, cytoplasmic retention is described; quantitative duration/serum stability NV.	HeLa cells: 0.05 wt/v% copolymer 9 in 5% glucose, room temperature 10 min, then wash; pH clamp with 20 μM nigericin. No animal injection (NA).
pDA/PEG NIR-II [4]	Standard pDA-5k-PEG: DLS mean 17.8 nm in PBS at measurement dilution; injected-concentration DLS NV. Dry AFM mean 2.9 nm; separately tuned DLS < 6 nm. pDA Mn 16.192 kDa, Mw 30.991 kDa, chain Đ 1.91 (THF GPC, polystyrene calibration).	Intrinsic polymer emission; dye-loading EE NA. Preparation: 75 µg/mL pDA in THF and 1.1 mg/mL DSPE-mPEG(5 kDa) in water mixed at 1:9 *v*/*v*. Recovered pDA:surfactant composition NR in examined main/SI.	Continuous 808 nm illumination for 1 h in PBS/FBS; <10% serum photobleaching. This tests emission, not colloidal size stability.	Tail-vein 200 µL, 0.25 mg/mL pDA-PEG in PBS.
FLAP networks [30]	NA nanoparticle DLS/TEM: bulk polymer specimens. NA finite Mn/Mw for the crosslinked polyurethane network. PTHF precursor Mn ~650 g/mol is not network Mn. Linear-polycarbonate Mn/Mw/Đ NV.	PC example: 0.05 wt% FLAP1. PU1/PU2, 10% TEA formulation: 0.20 mg FLAP/g polymer. Covalent probe content; encapsulation EE NA.	PU thermal conditioning: semicrystalline after >2 days at 25 °C; rubbery after 60 °C for 1 min. This is conditioning, not aqueous/serum stability; systemic stability NA.	NA: bulk polymer-material tensile experiments.
Loop-additive hydrogel [31]	NA nanoparticle diameter: macroscopic hydrogels. pOEGMA-Loop Mn 35 kg/mol, chain Đ 2.3; reference pOEGMA-NDI 32 kg/mol/2.3 and pOEGMA-BPEA 43 kg/mol/2.5.	Additive 0.03 wt/v% in hydrogel precursor; AAm 20 wt/v%, MBA 0.05 wt/v%. One central loop per proposed additive architecture. Encapsulation EE NA.	12 stretch/release cycles to 430% strain; relaxation at 380% strain followed 1600 s. These test reversible mechanochromism, not serum/colloidal stability (NA).	NA: bulk-hydrogel mechanical study.
RotAnBP hydrogel [32]	NA nanoparticle DLS/TEM; dry-film thickness 70–100 μm. Final linear polyurethane-urea Mn 48 kg/mol, chain Đ 2.04. PEG precursor Mn 2 kg/mol is not final-polymer mass.	RotAnBP feed 0.004 mol%; actual retained wt% NV. Covalent mechanophore incorporation; encapsulation EE NA.	Equilibrium water content 70% after 2 h in deionized water at room temperature; water content (%) = 100 × (M − M0)/M (M: swollen-film mass; M0: dry-film mass). Ambient drying ~0.5%/min initially; not demonstrated long-term hydrated stability.	NA: macroscopic hydrogel mechanical tests, no injection.

We exclude the polyrotaxane drug-loading percentage because the Results label, Methods denominator and stated feed are inconsistent; the percentage is not an IR783-loading value [7]. The previously characterised brush size is not a new DLS measurement of the injected rotor formulation [6]. We do not assign the separate always-ON dye content to turn-ON iP5B5 [28]. The two ASN/AIN particle populations and their chromophore-normalised doses are kept separate [42]. Values described as modelled, theoretical, feed or relative are not presented as measured detection limits, retained loading or absolute modulus.

**Table 3:** Controls that distinguish fluorescence changes from the biological processes inferred from them.

Optical Observation	Alternative Explanation	Discriminating Experiment
Higher tumour intensity	More reporter, altered tissue attenuation, free dye or phagocyte uptake	Matched acquisition and exposure; stability/free-dye controls; compartment-resolved localisation
Higher activation ratio	Different distributions of reporter and reference, spectral cross-talk or differential clearance	Characterise both modules; unmix spectra; verify the target with an independent assay
Longer rotor lifetime	Aggregation, compartment transfer or another quenching change	Formulation-specific calibration; decay analysis; localisation and environmental controls
Oxygen-sensitive phosphorescence lifetime	Cellular quenching, compartment mixing or an uncalibrated probe environment	Intracellular oxygen calibration; localisation; independent oxygenation measurement
Afterglow intensity or decay	Different stimulus history, oxygen/reactive-species availability or substrate consumption	Matched pre-activation and delay; stable control formulation; substrate and environmental calibration
Force-dependent fluorescence	Changed ligand density, anchoring, swelling or polymer organisation	Non-load-bearing control; characterised geometry; mechanical loading and unloading calibration
Signal after therapeutic irradiation	Bleaching, heating, photochemistry or probe-induced injury	Probe-only, light-only and treatment-component controls; independent temperature and viability measurements
Association with tumour response	Signal reflects cell death or changed tissue composition after regression	Early time points, longitudinal design, independent physical and functional endpoints, external validation

## Data Availability

No new data were created or analyzed in this study. Data sharing is not applicable to this article.

## Abbreviations

AFM	Atomic force microscopy
BODIPY	Boron-dipyrromethene
CAF	Cancer-associated fibroblast
DLS	Dynamic light scattering
DNA	Deoxyribonucleic acid
ECM	Extracellular matrix
FEM	Finite-element modelling
FLIM	Fluorescence lifetime imaging microscopy
FRET	Förster resonance energy transfer
GPC	Gel permeation chromatography
Gzmb	Granzyme B
ICG	Indocyanine green
IDO	Indoleamine 2,3-dioxygenase
NAD(P)H	Reduced nicotinamide adenine dinucleotide (phosphate)
NIR-II	Second near-infrared window
NO	Nitric oxide
NSET	Nanometal surface energy transfer
PAMAM	Poly(amidoamine)
PDI	Polydispersity index
PDT	Photodynamic therapy
PEG	Polyethylene glycol
pHPMA	Poly[N-(2-hydroxypropyl)methacrylamide]
PLGA	Poly(lactic-co-glycolic acid)
PLIM	Phosphorescence lifetime imaging microscopy
RAFT	Reversible addition–fragmentation chain transfer
ROS	Reactive oxygen species
SI	Supporting information
STING	Stimulator of interferon genes
TADF	Thermally activated delayed fluorescence
TAZ	Transcriptional coactivator with PDZ-binding motif
TEM	Transmission electron microscopy
TIRF	Total internal reflection fluorescence
UPS	Ultra-pH-sensitive
YAP	Yes-associated protein
